# DDX41 haploinsufficiency causes inefficient hematopoiesis under stress and cooperates with p53 mutations to cause hematologic malignancy

**DOI:** 10.1038/s41375-024-02304-9

**Published:** 2024-06-27

**Authors:** Emily Stepanchick, Andrew Wilson, Analise M. Sulentic, Kwangmin Choi, Kathleen Hueneman, Daniel T. Starczynowski, Timothy M. Chlon

**Affiliations:** 1 Division of Experimental Hematology and Cancer Biology, Cincinnati Children’s Hospital Medical Center, Cincinnati, OH USA; 2 Department of Pediatrics, University of Cincinnati, Cincinnati, OH USA; 3 Department of Cancer Biology, University of Cincinnati, Cincinnati, OH USA; 4 University of Cincinnati Cancer Center, Cincinnati, OH USA

**Keywords:** Cancer genetics, Haematopoiesis, Myelodysplastic syndrome

## Abstract

Germline heterozygous mutations in DDX41 predispose individuals to hematologic malignancies in adulthood. Most of these DDX41 mutations result in a truncated protein, leading to loss of protein function. To investigate the impact of these mutations on hematopoiesis, we generated mice with hematopoietic-specific knockout of one Ddx41 allele. Under normal steady-state conditions, there was minimal effect on lifelong hematopoiesis, resulting in a mild yet persistent reduction in red blood cell counts. However, stress induced by transplantation of the Ddx41^+/−^ BM resulted in hematopoietic stem/progenitor cell (HSPC) defects and onset of hematopoietic failure upon aging. Transcriptomic analysis of HSPC subsets from the transplanted BM revealed activation of cellular stress responses, including upregulation of p53 target genes in erythroid progenitors. To understand how the loss of p53 affects the phenotype of Ddx41^+/−^ HSPCs, we generated mice with combined Ddx41 and Trp53 heterozygous deletions. The reduction in p53 expression rescued the fitness defects in HSPC caused by Ddx41 heterozygosity. However, the combined Ddx41 and Trp53 mutant mice were prone to developing hematologic malignancies that resemble human myelodysplastic syndrome and acute myeloid leukemia. In conclusion, DDX41 heterozygosity causes dysregulation of the response to hematopoietic stress, which increases the risk of transformation with a p53 mutation.

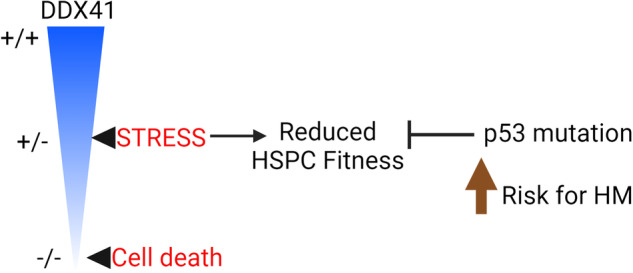

## Introduction

Heterozygous germline mutations in DDX41 cause predisposition to hematologic malignancies with a 50% lifetime penetrance [1]. Occurring in 2–4% of adult myelodysplastic syndrome (MDS) and acute myeloid leukemia (AML) cases, these mutations represent the most common cause of inherited predisposition to these diseases [1–6]. These mutations are typically frameshifts or affect the translation start site, resulting in loss of full-length protein expression [6]. However, the precise mechanisms by which they promote malignancy remain poorly understood [7].

In a recent study, we investigated the impact of inducible deletion of Ddx41 in bone marrow (BM) hematopoietic cells [8]. Deletion of both Ddx41 alleles in transplanted BM caused cell cycle arrest and death of hematopoietic progenitor cells (HPC), resulting in rapid BM failure. In contrast, mice with one functional Ddx41 allele in their BM remained healthy for up to one year but eventually exhibited symptoms of BM failure after 15 months. This suggests that DDX41 heterozygosity, akin to the germline state in patients, leads to an age-related decline in hematopoietic efficiency. However, this condition was not universally fatal and did not significantly affect survival compared to control mice, indicating that additional environmental or genetic factors are necessary to drive malignancy.

Germline DDX41-mutated MDS/AML cases exhibit a low mutation burden compared to general cohorts, typically with fewer than 2 co-mutations in cancer-associated genes [1, 2]. The most common somatic co-mutation affects the non-mutated allele of DDX41 and often causes the amino acid substitution R525H. This mutation is notably rare in general MDS/AML cohorts [1]. Our research revealed that HPC with one knockout DDX41 allele and one R525H-mutated allele undergo cell cycle arrest and apoptosis, suggesting that the acquired DDX41 mutation is not proliferation-driving [8]. Other commonly observed co-mutations in these patients include ASXL1, DNMT3A, CUX1, and TP53 [1, 2, 4, 5, 9, 10].

DDX41 has several described functions, including RNA splicing, R-loop resolution, snoRNA processing, ribosome biogenesis, and activation of innate immune signaling [7, 11]. However, it remains unclear how heterozygosity of DDX41 contributes to disease onset since defects in these functions have not been observed in DDX41-heterozygous cells.

In this study, we describe a comprehensive characterization of DDX41-heterozygous HSPC, which revealed HSPC fitness deficits during stress that could select for maladaptive mutations and led to the development of a highly penetrant model of DDX41-mutated MDS/AML.

## Methods

### Mice

Ddx41^flox^ mice were described previously [8]. These mice were bred to Rosa26-CreERT2 (Jackson #008463) or Vav-iCre (Jackson #008610) for inducible or hematopoietic-specific Cre activity. Trp53-knockout mice were obtained from Jackson (# 002101) [12]. All animal studies were conducted on an IACUC-approved protocol (2022-0055).

For BM transplant, 1e6 BM mononuclear cells (BMNC) were suspended in PBS and injected into the tail vein of lethally-irradiated recipient BoyJ mice. For competitive BM transplant, BMNC were mixed 1:1 with a total of 1e6 cells injected. For Rosa26-CreERT2 activation, 1 mg of tamoxifen dissolved in 50 µl corn oil was injected intraperitoneal daily for 5 days. Injections were repeated one week later.

### Flow cytometry

BMNC were stained with antibody cocktails in PBS containing 2% FBS. For analysis of HSPC, BMNC were stained with Mouse Hematopoietic Lineage Biotin Panel (eBiosciences), washed, and then fluorescent-tagged antibody cocktail containing streptavidin-e450.

For measurement of γH2AX, cells were fixed in 4% paraformaldehyde, washed, permeabilized in 90% cold methanol for 10 min, washed in Perm/Wash buffer (BD), and then stained with AF647-tagged antibody (BD)1:10 in Perm/Wash. Following a wash, samples were stained in Perm/Wash containing 2.5 µg/ml DAPI and then analyzed.

### In vivo measurement of protein synthesis and cell cycle

200 µL of a 10 mM solution of O-propargyl-puromycin (OP-puro) or 50 mg/mL solution of EdU (5-ethynyl 2´-deoxyuridine) was administered via intraperitoneal injection. BM was harvested after 2 h. BMNC were stained with antibodies, fixed in 1% paraformaldehyde, and then permeabilized in 0.1% saponin. An azide-alkyne cyclo-addition was performed using the Click-iT Cell Reaction Buffer and AlexaFluor-647 dye (Life Technologies) for 30 min. Cells were washed and then analyzed as described [13].

### Colony assay

Lineage-negative cells were isolated with EasySep Mouse Hematopoietic Progenitor Isolation Kit (STEMCELL). LSK cells were isolated by sorting. Cells were plated at 1000 Lin^−^ cells or 100 LSK cells per well in triplicate in MethoCult (STEMCELL) M3434 (myeloid) or M3436 (erythroid).

### CRIPSR knockout cell lines

Knockout cells lines were generated by transfection with Cas9:gRNA complexes using the NEON transfection system. See supplement for parameters.

### Erythroid differentiation culture

Lin^−^ cells were seeded in fibronectin-coated plates in IMDM (Gibco) supplemented with 10units/ml EPO, 10 ng/ml SCF, 10 μM dexamethasone (Sigma), 15% FBS, 1% detoxified BSA, 200 μg/ml holotransferrin (Sigma), 10 μg/ml recombinant human insulin (Sigma), 2mM L-glutamine, 10^−4^M β-mercaptoethanol and penicillin-streptomycin. After 48 h the medium was replaced by IMDM supplemented with 20% FBS, 2 mM L-glutamine and 10^−4^M β-mercaptoethanol. Cultures were analyzed on day 5, similarly to previous studies [14].

### RNA-sequencing

RNA was isolated using Quick RNA MiniPrep Kit (Zymo). Libraries were prepared with polyA selection using the TruSeq RNA Library Prep Kitv2 (Illumina) and then sequenced on a HiSeq2500. Reads were mapped to the reference genome, and then analyzed using iGeak [15] and AltAnalyze [16].

### Single-cell RNA-seq

BMNC were harvested 8wks post-transplant. Lin^−^cKit^+^ cells were sorted and stained by BioLegend TotalSeq-B multiplexing kit. Cells from 3 mice were mixed and 15,000 total nucleated cells were loaded onto 10X Chromium. Refer to supplement for 10X and sequencing parameters [17–21].

### Statistical analysis

All quantified assays were completed three times as biological replicates. Pairwise comparisons were conducted by Student’s *t* test. Statistical significance cutoff was *p* < 0.05 and denoted by an asterisk. Sizes of transplant groups were determined based on statistical power calculations using results from previous transplant studies. Mice who died prior to engraftment (28 days) were excluded. Littermate mice were used for transplant studies and were not randomized by any particular method. Investigators were not blinded to the identity of the mice. Error-bars represent standard deviations about the means. Sample sizes are denoted by the number of datapoints in each graph.

## Results

### Heterozygous loss of Ddx41 in hematopoietic cells causes mild anemia

**Fig. 1:**
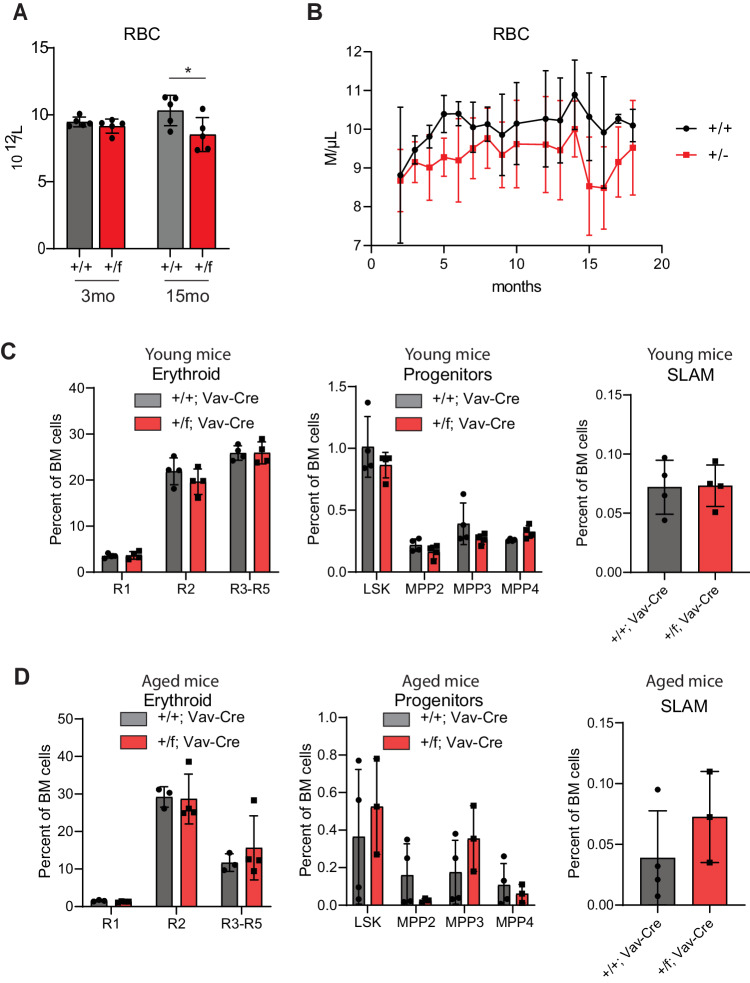
Mice with heterozygous loss of Ddx41 in the hematopoietic system are viable with mild reduction in red blood cells over the course of life. **A** Measurement of red blood cell concentration in peripheral blood at 3mo and 15mo of age. **B** Monthly measurement of red blood cell concentration in peripheral blood of mice (*n* = 5). **C**, **D** Flow cytometry for erythroid progenitors (CD71/Ter119) and hematopoietic stem and progenitor cells in bone marrow of young mice (8–12 weeks old) and old mice (24–26 months). The percentage of total cells within each gate is graphed.

To examine the effect of Ddx41-deficiency on hematopoiesis, we crossed conditional Ddx41-knockout mice with Vav-Cre mice (Ddx41^f/f^;Vav-Cre). Across many litters, we never observed a Ddx41^f/f^;Vav-Cre+ mouse, indicating that homozygous loss of Ddx41 in hematopoietic cells is embryonic lethal (Supplementary Fig. 1A). We examined E12.5 fetuses and found that the Ddx41^f/f^;Vav-Cre+ fetus was already not viable, whereas Ddx41^+/f^;Vav-Cre+ fetuses appeared normal and were born at normal Mendelian ratios (Supplementary Fig. 1B, C). This observation differs slightly from a previous report, where the Ddx41^f/f^;Vav-Cre+ mice died shortly after birth, although different but highly similar Ddx41^flox^ strains were used [22]. To determine the effect of Ddx41 heterozygosity, we observed a cohort of Ddx41^+/f^ ;VavCre+ mice and their littermates for over 2 years and did not observe a significant difference in survival (Supplementary Fig. 1D). Through monthly bleeding, we tracked their common blood counts (CBC) (Supplementary Fig. 1E). The only significant difference observed in Ddx41^+/f^;Vav-Cre+ mice was a consistent reduction in red blood cell (RBC) count across the lifespan that did not meet the criteria for clinical anemia (Fig. 1A, B). We did not observe any differences in the abundance of erythroid progenitors or in the proportion of HSPC subsets in young or aged mice (Fig. 1C, D). The cellularity and spleen size were also unaffected (Supplementary Fig. 1F).

We previously assessed the effect of Rosa-CreRT2-mediated inducible knockout of one Ddx41 allele after BM transplantation and found that there was an age-dependent decline in hematopoietic function that ultimately caused anemia and BM failure [8]. We confirmed that there is a reduction in Ddx41 protein in the BM HPC in this model and analyzed CBC data to show that there is consistent life-long reduction in RBC count and hematocrit that worsens with age (Supplementary Fig. 2A–D). These results indicate that DDX41 heterozygosity causes mild defects in steady state hematopoiesis that are restricted to the erythroid lineage, whereas more profound dyshematopoiesis phenotypes arise in the BM transplant setting with aging.

### Heterozygous loss of Ddx41 causes erythroid progenitor defects ex vivo

**Fig. 2:**
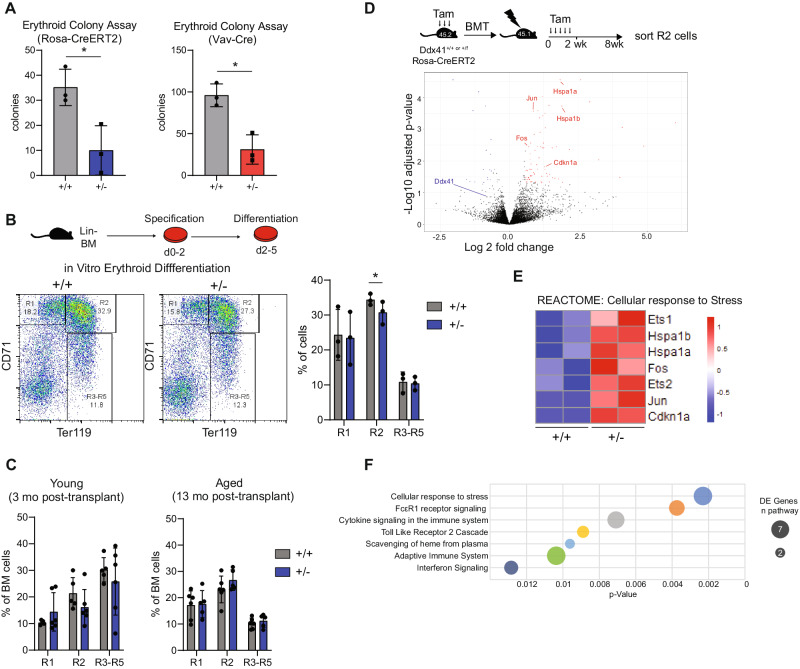
Ddx41^+/−^ erythroid progenitors have cell growth defect ex vivo and activated cell stress-related gene expression in vivo. **A** Erythroid colony formation in methylcellulose by Lin^−^ bone marrow cells from young mice of the indicated genotypes. **B** Ex vivo erythroid differentiation of Lin^−^ bone marrow cells in liquid culture. The percentage of erythroid progenitors in each gate on the CD71/Ter119 flow cytometry assay is graphed. **C** Flow cytometry for erythroid progenitors by CD71/Ter119 in bone marrow of mice transplanted with cells of the indicated genotype. **D** Volcano plot for differential gene expression in RNA-Seq on R2 (CD71+ Ter119+) erythroid progenitors from the bone marrow of transplanted mice at 8wks post-transplant. **E** Heatmap of differentially expressed genes in the Cellular Response to Stress pathway from the Reactome database. **F** Enrichment of Reactome pathways in differentially expressed genes from RNA-Seq on R2 cells.

To characterize the effect of Ddx41-heterozygosity on erythropoiesis, we examined the erythroid differentiation capacity of lineage-negative (Lin^−^) BM cells from Ddx41^+/+^ and Ddx41^f/+^ mice expressing either Rosa-CreRT2 or VavCre. In erythroid colony-formation assays, we observed reduced colony formation by Ddx41^+/−^ Lin^−^ cells (Fig. 2A). In liquid-culture differentiation assays, fewer erythroid progenitors were produced from Ddx41^+/−^ Lin^−^ cells (Fig. 2B). These results suggest that the production of erythroid-specified progenitors is delayed and/or blunted in Ddx41^+/−^ HSPCs. However, we did not observe significant differences in the abundance of erythroid progenitors in the BM of Ddx41^+/−^ mice, suggesting that these phenotypes are exacerbated by ex vivo conditions (Fig. 2C). To examine the effect of Ddx41-heterozygosity on in vivo erythroid progenitors, we sorted CD71^+^,Ter119^+^ cells (R2) from the BM of recipient mice 8-weeks post-transplant with Ddx41^+/f^;RosaCreERT2 BM. This timepoint was chosen to capture the cells in a highly proliferative state since the BM is still being replenished at this stage. We confirmed that the Ddx41^+/−^ R2 cells express less Ddx41 protein than Ddx41^+/+^ cells (Supplementary Fig. 3A). By transcriptome analysis, we found that Ddx41^+/−^ R2 cells have activated cellular stress signaling, including the early response genes (*cFos* and *Jun*), heat-shock response factors (*Hspa1a* and *Hspa1b*), and the cyclin-dependent kinase inhibitor *Cdkn1a* (Fig. 2D, E, Supplementary Fig. 3B). Pathway analysis of the differentially-expressed genes (DEGs) revealed enrichment of “cellular response to stress” and innate immune signaling-related pathways (Fig. 2F). Gene set enrichment analysis (GSEA) additionally revealed that ribosomal and protein translation initiation pathways are enriched (Supplementary Fig. 3C). Since Ddx41-heterozygosity affected cell cycle and ribosome-related genes, we determined its impact on the cell cycle using EdU-labeling and on protein translation using OP-puromycin (OPP) labeling in R2 cells. However, our findings did not reveal any significant differences (Supplementary Fig. 3D). We also examined DNA damage levels by staining for γH2AX but did not observe any discernible variation (Supplementary Fig. 3D). Furthermore, we investigated the effect on alternative splicing through RNA-Seq data analysis, but we did not detect substantial differences (Supplementary Fig. 3E). In summary, over-activation of cellular stress pathways occurs in Ddx41^+/−^ erythroid progenitors, leading to stress-activated deficiencies in erythropoiesis.

### Heterozygous loss of Ddx41 causes reduced stem cell engraftment activity

**Fig. 3:**
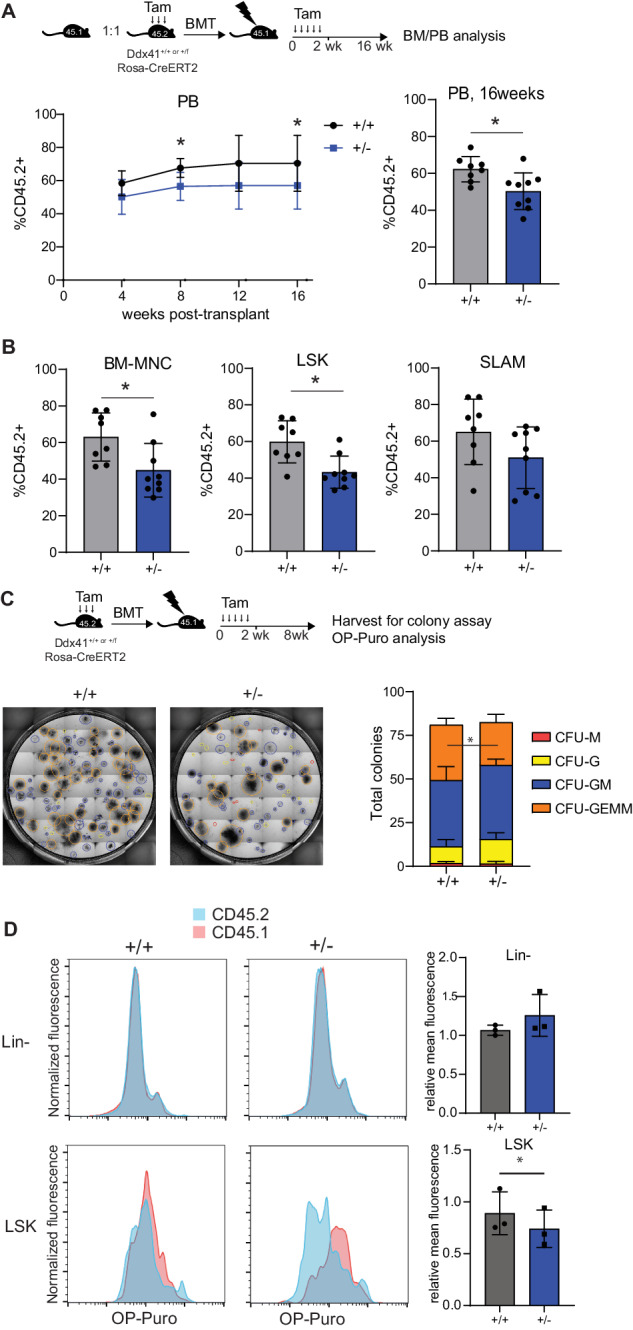
Ddx41^+/−^ hematopoietic stem and progenitor cells have modestly reduced fitness, correlating with reduced protein translation activity. **A** Competitive transplant of bone marrow cells from Ddx41^+/f^;Rosa-CreERT2 and control mice against CD45.1+ competitor bone marrow cells. Peripheral blood chimerism was determined by flow cytometry for CD45.2 and CD45.1 for 16 weeks. **B** Chimerism in bone marrow cell populations determined by flow cytometry for CD45.2 and CD45.1. **C** Non-competitive transplant of bone marrow cells from Ddx41^+/f^;Rosa-CreERT2 and wild-type Rosa-CreERT2 mice was conducted, and Lin^−^Sca^+^Kit^+^ cells were harvested for colony formation assays in methylcellulose. **D** In vivo staining for protein translation rate by OP-Puro injection into transplanted mice in **C** followed by flow cytometry on bone marrow cells. Relative OP-puro signal in each cell population is graphed.

To examine the effect of Ddx41-heterozygosity on HSPC function, we conducted competitive BM reconstitution assays using CD45.2 + BM cells from Ddx41^+/+^ or Ddx41^+/f^ mice expressing either Rosa-CreRT2 or VavCre. We used wildtype CD45.1 + BM cells as competitor cells. In both Cre systems, we observed a consistent yet modest reduction in engraftment by Ddx41^+/−^ cells in the peripheral blood (PB) throughout the duration of the experiment and in the BM at 16-week post-transplant (Fig. 3A, B and Supplementary Fig. 4A–C). Notably, this engraftment difference became apparent as early as 4 weeks post-transplant but did not intensify over time, suggesting that the difference occurs during the initial engraftment phase. We did not detect any distinctions in homing. In secondary transplants, there was significant variability in the BM chimerism across both groups, and thus there was no significant difference (Supplementary Fig. 4D–I). It is possible that the primary transplant selects for HSPC with optimized Ddx41 expression levels or other resistance to decreased Ddx41 expression, causing the effect to be lost in the secondary transplant.

To assess the cause of the reduced engraftment of Ddx41^+/−^ cells, we sorted CD45.2 + LSK cells from the transplanted mice 6-weeks post-transplant and examined HSPC colony formation potential (Fig. 3C). We found that Ddx41^+/−^ LSK cells formed similar numbers of colonies as compared to colonies formed by wildtype LSK; however, the Ddx41^+/−^ LSK cells produced fewer CFU-GEMM, which are formed from highly proliferative, multipotent HSPC. This reduction in the number of highly proliferative HSPC is consistent with the reduced engraftment observed in the competitive transplant.

To determine if the ribosome biogenesis function of Ddx41 is involved in this phenotype, we measured the protein translation rate in BM cells isolated from mice 8 weeks post-transplant by in vivo OP-puromycin incorporation. We observed reduced protein translation in Ddx41^+/−^ LSK cells as compared to wildtype LSK cells. Interestingly, the protein translational rates were comparable between Ddx41^+/−^ and wildtype BM Lin^−^ cells, suggesting these effects are confined to HSPC with a higher degree of stemness (Fig. 3D).

**Fig. 4:**
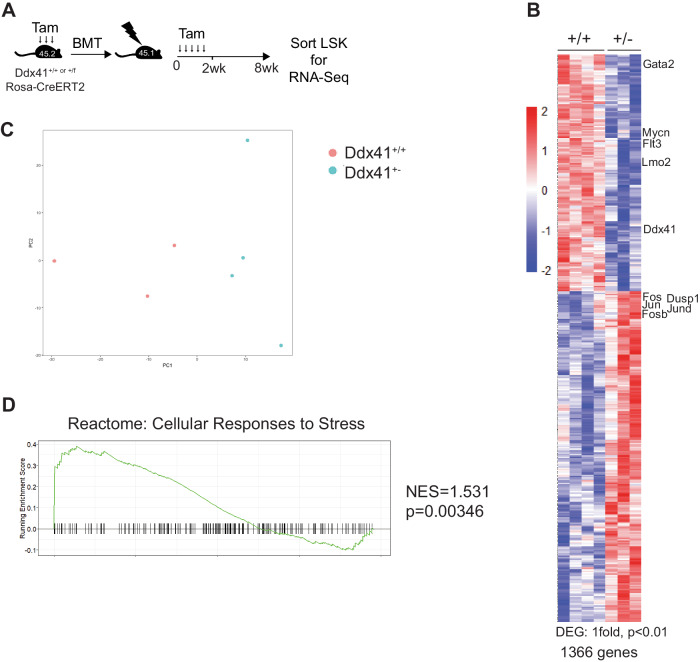
Transcriptome analysis in Ddx41^+/−^ hematopoietic stem and progenitor cells indicates activation of cellular stress pathways. **A** Experimental setup and tamoxifen-injection schedule for RNA-Seq on Lin^−^Sca^+^Kit^+^ cells from non-competitive transplant mice. **B** Heatmap of differentially expressed genes in Lin^−^Sca^+^Kit^+^ cells harvested from transplanted mice at 8wks post-transplant. **C** Principle component analysis on RNA-Seq samples for Lin^−^Sca^+^Kit^+^ sorted from Ddx41^+/f^;RosaCreRT2 bone marrow 6-weeks post-tamoxifen treatment. **D** Gene set enrichment analysis for the Cellular Response to Stress Reactome pathway.

To further characterize the mechanisms underlying the impaired phenotype of Ddx41^+/−^ HSPCs, we profiled the transcriptome of LSK cells isolated from recipient mice 8 weeks post-transplant. We observed significant gene expression changes in 1366 genes (Fig. 4A, B), and the samples clustered separately from one another in principle component analysis (Fig. 4C). Pathway analysis revealed enrichment for cellular stress and innate immune-related signaling pathways (Fig. 4D, Supplementary Fig. 5A, B). We also conducted single-cell RNA-Seq on sorted Lin^−^, c-Kit+ (LK) cells isolated from recipient mice 8 weeks post-transplant. We sequenced ~2500 LK cells from 3 independent mice of each genotype and then conducted unbiased cell clustering (Supplementary Fig. 5C) We named the cell clusters based on the expression of lineage-specific genes using a single-cell atlas of mouse hematopoiesis [23] (Supplementary Fig. 5D). We did not observe significant changes in the abundance of cells in any of the clusters, although there was a trend towards increased granulocyte-biased progenitors and reduced megakaryocyte-erythrocyte-biased progenitors (Supplementary Fig. 5E, F). We also did not observe any significant changes in gene expression (log_2_(FC) > 0.25) within any of the cell populations. The lack of significant differences are likely due to the reduced depth of sequencing in scRNA-Seq compared to bulk RNA-Seq. We assessed the expression of Ddx41 and found that its expression level was reduced but did not meet the significance threshold in any of the cell clusters (Supplementary Fig. 5G). Collectively, the transcriptomic data indicate that heterozygosity of Ddx41 causes activation of cellular stress pathways in HSPCs, potentially through reduced ribosome function, but that this stress does not substantially alter the hematopoietic output of the progenitor cells in vivo at steady state. In conditions requiring rapid and/or extensive cell proliferation, there is a relative decline in stem cell fitness that manifests as reduced engraftment in competitive transplant and reduced colony formation.

### TP53 loss-of-function mutations co-occur with germline DDX41 mutations and rescue the DDX41-mutant cellular defects

**Fig. 5:**
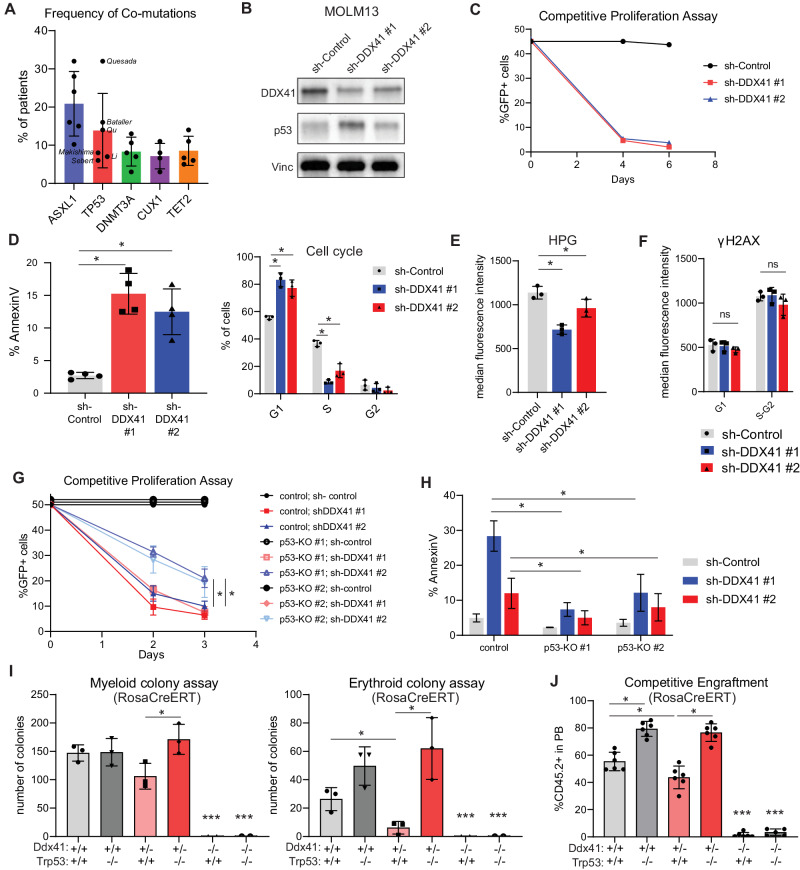
Reduced DDX41 expression causes p53 activation and the effects of incomplete loss of DDX41 can be partially rescued by p53 loss. **A** The percentage of germline DDX41 MDS/AML patients with co-mutations in the indicated genes from five different patient cohort studies. **B** Western blot for DDX41 and p53 expression in MOLM13 cells expressing anti-DDX41 shRNAs. **C** The percentage of GFP+ cells in 1:1 mixed cultures of MOLM13 cells transduced with sh-control or sh-DDX41 and non-transduced MOLM13 cells. The vector for shRNA expression is pLKO.1-GFP. **D** Flow cytometry for AnnexinV and cell cycle in cultures of MOLM13 cells expressing anti-DDX41 shRNAs. **E** Measurement of protein translation activity by flow cytometry for HPG incorporation in MOLM13 cells expressing anti-DDX41 shRNAs compared to sh-control. **F** Measurement of DNA damage by flow cytometry for γH2AX in MOLM13 cells expressing anti-DDX41 shRNAs compared to sh-control. **G** The percentage of GFP+ cells in 1:1 mixed cultures of MOLM13 cells transduced with sh-control or sh-DDX41 cells and non-transduced cells. **H** Flow cytometry for AnnexinV in cultures of p53-knockout MOLM13 cells expressing anti-DDX41 shRNAs. **I** Colony formation from Lin^−^ cells of the indicated genotypes in methylcellulose containing 4-OHT and cytokines permissive of myeloid or erythroid colony growth. **J** Peripheral blood chimerism measured by flow cytometry for CD45.2 in mice transplanted with a 1:1 mixture of cells of the indicated genotype and control CD45.1 cells.

To understand how Ddx41 heterozygosity contributes to hematologic malignancy, we sought to model the disease by targeting genes where mutations are observed in patients. We analyzed published cohorts of DDX41-mutated MDS/AML patients and determined the frequency of common co-mutations in each cohort. Somatic DDX41 mutation was the most common, followed by ASXL1, TP53, DNMT3A, TET2, and CUX1 (Fig. 5A). Since we had observed activation of the p53 target gene *Cdkn1a* in Ddx41^+/−^ HSPCs, we sought to determine the contribution of p53 activity to hematologic disease in the context of Ddx41 mutations. To examine the effect of DDX41 loss on p53 activity, we knocked down DDX41 expression using shRNAs (shDDX41) in the MOLM13 AML cell line, which is wildtype for TP53. We used two different shRNAs, one that causes nearly complete loss of DDX41 (shDDX41 #1) and another that causes approximately 60% loss (shDDX41 #2). We observed increased p53 protein abundance upon loss of DDX41, and the more-effective shRNA (#1) caused the largest increase (Fig. 5B, Supplementary Figure 6A). Both DDX41-targeting shRNAs induced cell cycle arrest and apoptosis within 4 days of transduction (Fig. 5C, D, Supplementary Fig. 6B), and a significant reduction in protein translation, as measured by incorporation of the methionine-analog HPG (Fig. 5E, Supplementary Fig. 6C).

To further understand the basis of p53 activation upon DDX41 loss, we performed RNA-Seq on MOLM13 cells expressing anti-DDX41 shRNAs (Supplementary Fig. 6D). In shDDX41-#1-expressing cells, we observed activation of stress response gene signatures, similar to ones observed in Ddx41^+/−^HSPCs. However, the most significant pathway enrichment in cells expressing shDDX41-#1 were related to cell cycle regulation (Supplementary Fig. 6E, F). In contrast, cell cycle-related pathways were not enriched in the shDDX41-#2 DEGs, and instead innate immune signaling pathways were most enriched (Supplementary Fig. 6F). These findings suggest that complete and partial loss of DDX41 expression have distinct effects on cellular response. To gain further insight, we examined enrichment of transcription factor binding sites in the DEGs for each shRNA. This analysis revealed that target genes of E2F and other cell-cycle-related factors were enriched in DEGs from cells expressing shDDX41-#1, consistent with a strong cell cycle shutdown phenotype [24]. In contrast, for shDDX41-#2, we found transcription factors that are activated by innate immune signaling like IRF8 and RELA as well as p53 (Supplementary Fig. 6G). This indicates that incomplete loss of DDX41 is associated with innate immune and p53 pathway activation. We did not observe increased γH2AX staining with either DDX41-targeting shRNA, indicating that DNA damage is not the cause of the p53 induction and cell stress signaling (Fig. 5F, Supplementary Fig. 6H). To determine the role of p53 in the DDX41-deficient cellular lethality phenotype, we created p53-knockout MOLM13 cells by CRISPR-mediated gene editing (Supplementary Fig. 7A, B). Using two different clonal p53 knockout MOLM13 cell lines, we observed that p53 loss did not alter the protein translation deficit caused by either DDX41-targeting shRNA (Supplementary Fig. 7C), but it did partially rescue the proliferation and viability defect in cells expressing shDDX41-#2 (Fig. 5G, H). In contrast, the growth of shDDX41-#1 cells was not rescued despite a reduction in Annexin V positivity (Fig. 5G, H). Again, DNA damage levels measured by γH2AX staining were unaffected by loss of DDX41 (Supplementary Fig. 7D). Collectively, these findings indicate that p53 activation contributes to the cell death and cell cycle delay phenotype caused by partial reduction of DDX41 expression.

To determine the effect of p53-deficnency on the phenotype of Ddx41-deficienct mouse HSPCs, we crossed our Ddx41^flox^;Rosa-CreERT2 mice with Trp53-knockout mice. Lin^−^ BM cells were isolated from Ddx41^+/+^, Ddx41^+/f^, and Ddx41^f/f^ Trp53^−/−^ mice and examined in hematopoietic progenitor colony assays and competitive BM reconstitution. We found that deletion of Trp53 did not rescue the growth or engraftment of Ddx41^−/−^ hematopoietic cells (Fig. 5I). We also tested the effect of Trp53-knockout on the growth and engraftment defects in HSPC bearing combined knockout and R525H point mutation of Ddx41, which models the mutations observed MDS/AML patients, and did not observe any rescue (Supplementary Fig. 7E, F). In contrast, deletion of Trp53 increased colony formation and engraftment of Ddx41^+/−^ HSPCs, rescuing the fitness defect caused by Ddx41-heterozygosity (Fig. 5I, J). These data corroborate the findings in MOLM13 cells, indicating that p53 loss cannot rescue the effects of complete DDX41 loss but can rescue phenotypes resulting from partial loss of Ddx41.

### Heterozygous loss of p53 rescues HSPC phenotypes caused by heterozygosity of Ddx41

**Fig. 6:**
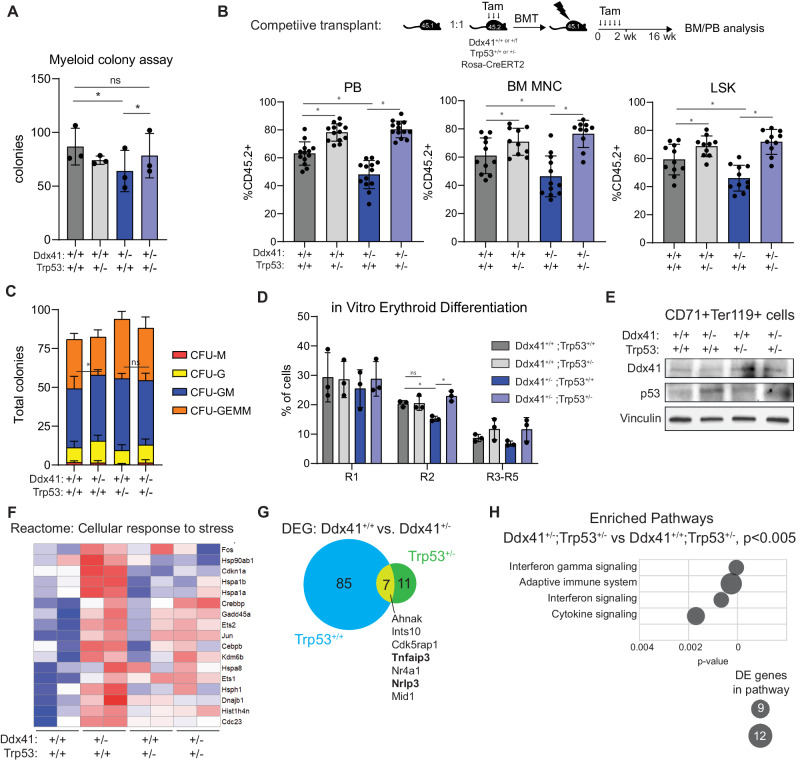
Heterozygous loss of p53 rescues reduced fitness phenotype of Ddx41^+/−^ hematopoietic stem and progenitor cells. **A** Myeloid colony formation from Lin^−^ cells of the indicated genotypes in methylcellulose containing 4-OH-Tamoxifen. **B** Competitive transplant of bone marrow cells from Ddx41^+/f^;Trp53^+/−^; Rosa-CreERT2 and control mice against CD45.1+ competitor cells. Chimerism of CD45.2+ cells measured by flow cytometry in the peripheral blood and bone marrow. **C** Colony formation in methylcellulose from Lin^−^Sca^+^Kit^+^ cells sorted from non-competitive bone marrow transplant mice at 8wks post-transplant. **D** Quantification of erythroid progenitors by flow cytometry on in vitro erythroid differentiation cultures of Lin^−^ bone marrow cells from Ddx41^/f^;p53^+/−^;RosaCreERT2 cells. **E** Western blot on erythroid cells from day5 of in vitro differentiation protocol. **F** Heatmap of expression for genes from the Cellular response to stress Reactome pathway in RNA-Seq on Lin^−^Sca^+^Kit^+^ cells. **G** Overlap of differentially expressed genes from RNA-Seq on Ddx41^+/+^ vs Ddx41^+/−^ Lin^−^Sca^+^Kit^+^ cells in Trp53^+/+^ vs Trp53^+/−^ context. 7 genes are differentially expressed in Ddx41^+/−^ Lin^−^Sca^+^Kit^+^ cells independent of Trp53 status. **H** Enrichment of Reactome pathways in differentially expressed genes from RNA-Seq on Ddx41^+/+^ vs Ddx41^+/−^ Lin^−^Sca^+^Kit^+^ cells from Trp53^+/−^ mice.

Given the highly oncogenic phenotype associated with Trp53 knockout, we sought to partially attenuate p53 activity and then evaluate its impact on HSPC function in Ddx41^+/−^ mice. To do this, we generated Ddx41^+/f^;RosaCreERT2 mice with heterozygous loss of p53 (Trp53^+/−^). Importantly, TP53 mutations in DDX41-mt MDS/AML patients tend to be heterozygous missense or frameshift mutations [1]. We initially conducted a colony assay and observed that Trp53 heterozygosity increased colony formation in Ddx41^+/−^ HSPCs, but it had no significant effect on Ddx41^+/+^ HSPCs (Fig. 6A). To determine the effect on HSPC function in vivo, we conducted a competitive BM transplantation assay and found that Trp53 heterozygosity overcomes the engraftment defect of Ddx41^+/−^ HSPCs in PB and BM (Fig. 6B). While it is known that Trp53-heteozygosity increases engraftment efficiency, we observe that the engraftment of Ddx41^+/+^;Trp53^+/−^ and Ddx41^+/−^:Trp53^+/−^ cells is indistinguishable. If the effect of the Trp53 heterozygosity was unrelated to the mechanism of reduced engraftment of the Ddx41^+/−^ cells, then we would expect the engraftment of Ddx41^+/−^:Trp53^+/−^ to be lower than Ddx41^+/+^;Trp53^+/−^. Since they are indistinguishable, we conclude that the reduction in Trp53 expression specifically rescues the HSPC fitness defect caused by Ddx41 heterozygosity. The effect of Trp53 heterozygosity on engraftment was accentuated by secondary BM transplantations in both groups and there was still no significant difference (Supplementary Fig. 8A). To evaluate the consequences of sustained p53 loss on the progenitor function of DDX41-mutant HSPCs, we also performed a colony assay on LSK cells isolated at 8-weeks post-transplantation. Heterozygous loss of Trp53 also restored the hematopoietic progenitor cell colony formation defect of Ddx41^+/−^ LSK (Fig. 6C, Supplementary Fig. 8B). These findings indicate that attenuated p53 function can rescue the deficit of Ddx41^+/−^ HSPC function in the BM.

To determine whether heterozygous loss of p53 also impacts erythropoiesis from Ddx41^+/−^ HPC, we performed in vitro erythroid differentiation assays. Trp53-heterozygosity rescued the formation of Ddx41^+/−^ CD71+/Ter119+ erythroid progenitors compared to wild-type levels (Fig. 6D, Supplementary Fig. 8C). We confirmed that Ddx41 levels were reduced in the Ter119+ cells from these cultures, and we found that p53 levels were elevated in Ddx41^+/−^ cells compared to wild-type cells (Fig. 6E). To further characterize the effects of combined Ddx41/Trp53 heterozygosity on erythroid progenitors in vivo, we sorted R2 BM cells 8-weeks post-transplant and performed gene expression analysis. In comparison to the 92 DEGs observed in Ddx41^+/−^ vs. Ddx41^+/+^ R2 cells when Trp53 was wild-type, we detected only 18 DEG when Trp53 was heterozygous. Notably, the “cellular response to stress” gene set, which was activated in Ddx41^+/−^ HSPC when compared to wild-type, showed a less pronounced activation in Ddx41^+/−^;Trp53^+/−^ cells (Fig. 6F). Seven genes were found to be differentially expressed in Ddx41^+/−^ R2 cells independent of Trp53 status, including Tnfaip3 and Nlrp3, both of which are involved in aspects of the innate immune response (Fig. 6G). Pathway analysis of the DEG in Ddx41^+/−^;Trp53^+/−^ compared to Ddx41^+/+^;Trp53^+/−^ cells revealed the presence of innate immune and cytokine-related signaling pathways (Fig. 6H). This suggests that innate immune activation persists in Ddx41^+/−^ cells even when Trp53 is heterozygous.

p53 is commonly activated by genotoxic stress but can also be activated in cells with ribosome biogenesis defects through increased abundance of free ribosomal proteins [25]. The mechanism for this regulation is that the free ribosomal proteins bind to MDM2 and cause it to release p53, reducing its proteasomal degradation. A mutant of Mdm2 (C305F) has reduced binding to free ribosomal proteins and is thus not affected by ribosome defects (Supplementary Fig. 8D) [26]. Thus, to determine if the activation of p53 that contributes to phenotypes of Ddx41^+/−^ HSPCs is caused by inefficiency of ribosome biogenesis, we crossed Ddx41^flox^ mice with Mdm2^C305F^mice. We conducted a competitive transplant assay with BM cells from Ddx41^+/f^;RosaCreERT2;Mdm2^C305F^ mice and controls (Supplementary Fig. 8E). As expected from previous reports, the Mdm2 mutation reduces BM chimerism in competitive transplants, which limited the potential for a specific rescue of the Ddx41^+/−^ phenotype [27]. Despite the reduced engraftment efficiency of the Mdm2-C305F cells, we observed that the presence of this mutation masked the Ddx41^+/−^ phenotype, such that there was no significant difference in engraftment caused by Ddx41-heterzygosity in the Mdm2 mutant cells. This result supports the conclusion that ribosomal stress through Mdm2/p53 contributes to the fitness defect of Ddx41^+/−^ stem cells.

### Combined heterozygosity of p53 and Ddx41 causes a highly penetrant hematologic malignancy

**Fig. 7:**
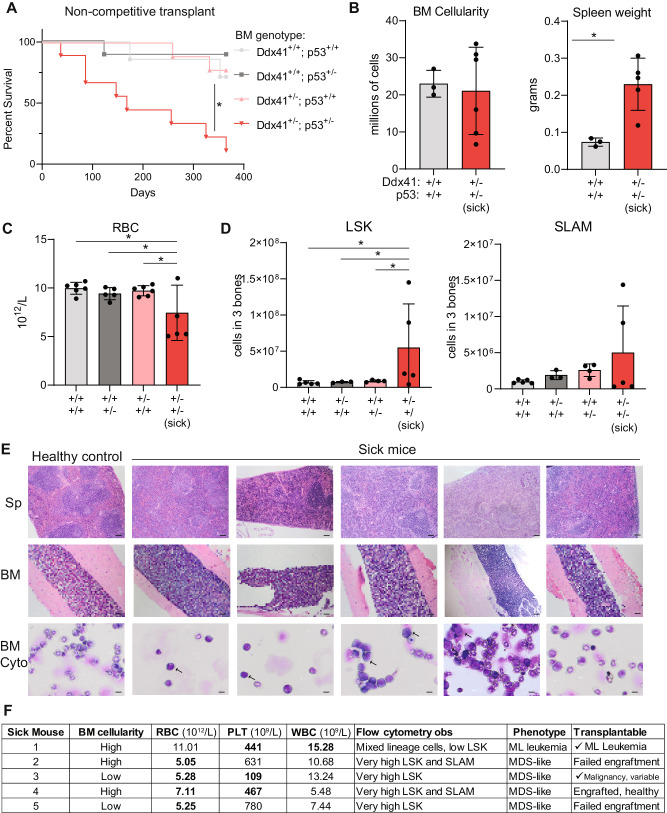
Combined heterozygosity of Ddx41 and p53 causes near-fully penetrant hematologic malignancy. **A** Kaplan–Meier plot for survival of mice transplanted with bone marrow of the indicated genotypes. **B** Bone marrow cellularity and spleen weight in mice at time of sacrifice (one year for controls). **C** Common blood count data for mice at one year-old or at time of sacrifice. **D** Quantitation of the number of Lin^−^Sca^+^Kit^+^ and SLAM (Lin^−^Sca^+^Kit^+^CD48^-^CD150^+^) cells in the bone marrow of sick mice compared to controls at end of experiment. **E** H&E stains of spleen and bone marrow sections and Wright-Geimsa stains of bone marrow cytospins from sick mice compared to a control group mouse. **F** Table indicating common blood count and other phenotypic data for sick mice from the Ddx41^+/−^;Trp53^+/−^ group.

To assess the effect of combined Ddx41 and Trp53 heterozygosity on the development of hematologic disease, we transplanted CD45.2+ Ddx41^+/f^;Trp53^+/−^;RosaCreERT2 BM cells into lethally-irradiated CD45.1+ recipients without competitor cells, excised the Ddx41-floxed allele by tamoxifen treatment, and then followed the recipient mice for up to one year. Within 3 months post transplantation, mice engrafted with Ddx41^+/−^;Trp53^+/−^ BM cells began to became moribund. By 12 months, 9 of 10 mice developed a hematologic disease (Fig. 7A). The hematologic phenotype was variable in that some mice had hypercellular BM whereas others were hypocellular. The spleens were universally enlarged due to extramedullary hematopoiesis or blasts (Fig. 7B). Most mice were anemic, whereas platelet and white blood cell numbers were variable (Fig. 7C, Supplementary Fig. 9A). Flow cytometry for HSPC in the BM revealed increased LSK and SLAM + LSK cell abundance in a portion of the Ddx41^+/−^;Trp53^+/−^ moribund mice (Fig. 7D, Supplementary Fig. 9B). The splenic architecture was abnormal in all mice, having reduced red pulp and increased abundance of nucleated cells throughout the tissue, consistent with extramedullary hematopoiesis (Fig. 7E). By flow cytometry on the spleen, we observed altered proportions of lymphoid/myeloid cell sets, including an expansion of myeloid cells (Supplementary Fig. 9C). We also observed abnormal architecture of the BM with overcrowding by nucleated cells in several of the sick mice (Fig. 7E).

To determine whether the hematologic phenotype is durable, we performed secondary transplants using Ddx41^+/−^;Trp53^+/−^ BM cells from independent sick mice. We observed engraftment by BM cells from 3 out of 5 sick mice, with 2 samples causing lethal hematologic malignancy phenotypes similar to their donor (Fig. 7F, Supplementary Fig. 10A). Recipient mice engrafted with Ddx41^+/−^;Trp53^+/−^ BM cells (from sick mouse #1) developed a rapid and lethal disease, which included expansion of Ddx41^+/−^;Trp53^+/−^ cells in the BM and PB (Supplementary Fig. 10A, B). Both donor (sick mouse #1) and recipients had blasts with mixed cellular phenotype (Gr1 + /CD3e + ) in the BM, spleen, and PB, and had very low numbers of LSK and SLAM (Supplementary Fig. 10C, E). The recipients were anemic, thrombocytopenic, and had an elevated WBC count, similar to the donor (Supplementary Fig. 10D). Recipient mice engrafted with BM cells from sick mouse #3 also developed a lethal condition. These mice exhibited hallmarks of MDS including thrombocytopenia and lymphopenia, and marked accumulation of CD45.2 + LK and LSK cells in the BM with modest CD45.2+ engraftment in the PB, indicating impaired differentiation (Supplementary Fig. 10D, E). Recipients of BM from sick mice #1 and #3 had enlarged spleens, abnormal splenic architecture, and blast-like cells in the BM (Supplementary Fig. 10F, G). Recipient mice engrafted with BM cells from three other diseased donor mice did not develop a lethal disease within the study period, due to low engraftment, which is common with models of MDS, or the presence of healthy donor HSC that outcompeted the diseased HSC upon transplant (sick mouse #4) (Fig. 7F, Supplementary Fig. 10A). To determine the presence of cooperating mutations in the diseased mice, we assessed the presence of the wild-type p53 allele and found that it was not lost in any of the mice at time of sacrifice (data not shown), and we also conducted whole exome sequencing on two mice (sick mice #3 and #4) and did not observe the acquisition of mutations in known leukemia-causing genes (Supplementary Table). Collectively, these data indicate that combined heterozygosity for Ddx41 and Trp53 drives a highly penetrant hematologic malignancy phenotype that is often characterized by stem cell expansion, anemia, and thrombocytopenia, and can transform into leukemia in some instances.

## Discussion

Several potential mechanisms have been proposed for how DDX41 mutations promote malignancy [6, 28–30]. Our models demonstrate that heterozygous loss of DDX41 is not itself an oncogenic driver since Ddx41-heterozygous mice live long lives with only mild hematologic phenotypes. The strong association of DDX41 mutation with low mutation burden and normal cytogenetics argues against genomic instability as the predominant mechanism of transformation in these patients. Accordingly, we did not observe increased DNA damage foci in DDX41-mutant cells. This observation differs from previous reports showing that DDX41 prevents R-loop formation and associated DNA damage [28, 29, 31]. It is possible that the apparent contradiction of these observations is caused by the cellular context or mode of inhibition of DDX41 expression used. Importantly, our analysis of DNA damage levels included in vivo hematopoietic progenitor cells with heterozygous loss of DDX41, precisely modeling the cellular context and DDX41 status of pre-disease cells. Nonetheless, our study does not rule out a role for DDX41 in maintaining genome integrity in some cellular contexts. It is also important to note that our analysis of the effect of partial reduction in DDX41 expression in leukemia cells (MOLM13 cells) was conducted in a transient setting, with analysis occurring 4–6 days post-transduction. A previous study showed that partial reduction in DDX41 expression promoted the leukemogenicity of K562 cells, but this experiment was conducted on selected cells over a longer time period, which may explain the discrepancy [6].

A recent study of DDX41 mutation carriers indicated that their blood counts are predominantly normal prior to disease onset, except for a mild decrease in RBC [32]. This condition is precisely replicated by conditional Ddx41^+/−^ mice, which are mildly anemic throughout their lifespan. This and the reduced output from in vitro erythroid differentiation cultures indicate that the efficiency of erythropoiesis is reduced by heterozygosity of Ddx41. A previous report also demonstrated that partial loss of ddx41 in zebrafish predominantly effects erythropoiesis [28]. We posit that the erythroid lineage is uniquely affected at steady state because erythropoiesis requires the highest rates of cellular proliferation among the blood lineages.

Since other blood cell lineages are not perturbed by Ddx41-heterozygosity during steady-state hematopoiesis, it is unlikely that overt hematopoietic failure is a mechanism for disease initiation in DDX41-mt patients. Rather, our data support the conclusion that DDX41-heterozygosity reduces HSPC fitness during times of rapid cellular proliferation, which we have modeled using BM transplantation stress. In the post-transplantation period, we observed increased cellular stress signaling in both LSK cells and erythroid progenitors, with evidence of activated p53 signaling in the erythroid cells. The TP53 tumor suppressor is a major regulator of cellular stress signaling, and thus we utilized TP53-heterozygosity to reduce this cell stress signaling while keeping the genome surveillance function of p53 intact. The rescue of Ddx41^+/−^ HSPC defects by reduced p53 expression indicates that cellular stress is a cause of the HSPC fitness defect, and that selective pressure for mutations that attenuate cellular stress signaling is heightened in Ddx41^+/−^ HSPC pools. In some cases, these mutations may be maladaptive, such as p53 mutations. Importantly, DDX41-mt MDS/AML patients bearing heterozygous p53 frameshift mutations have been observed in the clinic. The elderly age of disease onset in DDX41-mt patients is consistent with gradual selection of malignant clones through episodes of hematopoietic stress, such as infections.

While heterozygous loss of p53 reduces cellular stress signaling caused by DDX41-heterozygosity, it is unlikely to mitigate the source of the cellular stress, such as a ribosome defect. Frequently, cellular stress in the hematopoietic compartment activates inflammatory and innate immune response pathways, and these pathways are known drivers of MDS and AML pathogenesis [23, 33]. Importantly, we found that p53 heterozygosity reduced the cellular stress signaling but did not attenuate innate immune activation associated with Ddx41-heterozygosity, and it is likely that uncontrolled activation of these pathways contributes to the onset of malignancy in our model.

Multiple functions have been described for DDX41, including splicing, R-loop repair, and ribosome biogenesis, and reduced efficiency of any of these pathways could contribute to cellular stress. While our data support a role for inefficient ribosome biogenesis in causing the HSPC fitness defects, it is possible that inefficiencies in many of these cellular functions are involved. Future studies are necessary to identify targeted therapeutic strategies to rescue these cellular fitness defects such that selection of malignant clones in the bone marrow of patients can be prevented.

### Supplementary information

Supplementary Information

Supplemental Table 1

## Data Availability

All raw data are available by request from the corresponding author. Sequencing data has been deposited to the Gene Expression Omnibus (GSE248396 and GSE247409).
