# Supplementary material for: DDX41 haploinsufficiency causes inefficient hematopoiesis under stress and cooperates with p53 mutations to cause hematologic malignancy
Source: Leukemia. 2024 Jun 27;38(8):1787–98. doi: 10.1038/s41375-024-02304-9 (PMC11286521; doi:10.1038/s41375-024-02304-9)
Supplement: Supplementary file 1 — Supplementary Information [file 41375_2024_2304_MOESM1_ESM.pdf]

Supplementary Methods

Mice

Animals were bred and housed in the Association for Assessment and Accreditation of Laboratory Animal Care-accredited animal facility of Cincinnati Children's Hospital Medical Center. All mouse work was conducted under an IACUC-approved protocol (2022-0055). All mouse strains were kept on C57BL/6N background. When mice were moribund (pale, extreme lethargy, panting, etc), they were sacrificed by CO_2 asphyxiation, and bones and spleen were harvested for analysis. BM was harvested by crushing leg bones with a mortar and pestel. RBC were lysed in 1X Lysing Buffer (BD Biosciences). For longitudinal studies, mice were bled monthly by submandibular puncture to obtain 50-100µl of blood.

Cell Lines

MOLM13 cell lines were obtained from AddexBio Cat# C0003003 (Independently STR profiled to confirm identity in August 2023) cultured in RPMI 1640 medium with 20% FBS and 1% Penicillin-streptomycin. HEK293T cell line for virus production was cultured in DMEM with 10% FBS and 1% Penicillin-streptomycin.

CRIPSR Knockout Cell lines

To generate p53-knockout MOLM13 cells, we transfected cells with gRNA:Cas9 complexes using 1.5µg Alt-R S.p.Cas9 (IDT) with 1µg sgRNA (Synthego) in the NEON transfection system (Thermo, settings:1400V, 20ms, 1 pulse). Single cell clones were screened for loss of p53 by immunoblot and then confirmed by Sanger sequencing.

Flow Cytometry

Antibodies included B220, CD11b, Ly-6G, CD45.2, CD45.1, c-Kit, Sca-1, CD135 (eBiosciences); Ter119, CD71, CD3e, CD48, Streptavidin (Thermo); CD150 (Biolegend). For flow cytometry analysis of HSPC populations, the following gating was used: LSK (Lin^{neg}, $Sca\text{-}1^+$, $c\text{-}Kit^+$); LSK-SLAM (Lin^{neg}, $Sca\text{-}1^+$, $c\text{-}Kit^+$, $CD48^-$, $CD150^+$), MPP-2 (Lin^{neg}, $Sca\text{-}1^+$, $c\text{-}Kit^+$, $CD34^+$, $CD48^+$, $CD150^+$, $CD135^-$), MPP-3 (Lin^{neg}, $Sca\text{-}1^+$, $c\text{-}Kit^+$, $CD34^+$, $CD48^+$, $CD150^-$, $CD135^-$), and MPP-4 (Lin^{neg}, $Sca\text{-}1^+$, $c\text{-}Kit^+$, $CD34^+$, $CD48^+$, $CD150^-$ $CD135^+$). All live-cell flow samples were stained with 7-AAD (eBiosciences) for dead/dying cells immediately before analysis. For in vitro measurement of protein synthesis, Click-iT HPG Alexa-Fluor 594 Protein Synthesis Assay was used (Thermo).

Virus production

DDX41 shRNAs expressed from pLKO.1-GFP were generated previously[8]. Lentiviral supernatants (pLKO) were made by transfecting HEK293T cells using Trans-LT (Mirus) transfection reagent with lentiviral plasmid, pCDLN packaging vector, and pMD.2 VSV-G envelope vector and harvesting the culture medium 48hrs post-transfection. Cells were transduced with viral supernatant containing 0.8µg/ml polybrene for 24hrs. Expression of GFP was determined using flow cytometry.

Colony assays

For Rosa-CreERT2 colony assays, 1µM 4-hydroxytamoxifen was added to the methocult. Colonies were counted after 12 days using Stemvision (StemCell Technologies).

RNA-Sequencing Analysis

We obtained 20M 75bp paired-end reads. For splicing analysis of the RNA-Seq data, alternative splicing events were predicted using AltAnalyze (http://www.altanalyze.org, v2.1.3) with mouse mm10 Ensembl database (v72)[29]. Briefly, alternative gene/exon statistics were calculated using a unpaired moderated t-test option and default cutoffs (dabg_p (p-value corresponds to the detection above background (DABG)): 1.0, junction expression threshold: 5.0, exon_exp_threshold: 5.0, gene_exp_threshold: 200.0, exon_rpkm_threshold: 0.5, gene_rpkm_threshold: 1.0). To identify differential alternative exon usages and alternative splicing events, the MultiPath-PSI splicing algorithm was chosen with p-value cutoff < 0.05 (alt_exon_fold_variable: 0.1, gene_expression_cutoff: 10.0.

Single-cell RNA Sequencing

The scRNA-Seq assay was performed according to the manufacturer's instructions (Chromium Next GEM Single Cell 3' Reagent Kits v3.1 (Dual Index) with Feature Barcode technology for Cell Surface Protein, 10x Genomics). Briefly, Total-Seq B antibody-labeled cells were resuspended in the master mix and loaded together with partitioning oil and gel beads into the chip to generate a gel bead-in-emulsion (GEM). The poly-A RNA from the cell lysate contained in every GEM was reverse transcribed into cDNA, adding an Illumina TruSeq R1 primer sequence, Unique Molecular Identifier (UMI) and the 10x Barcode. The DNA conjugated to the Total-Seq B antibodies (Feature Barcodes aka FBs) was also barcoded by adding an Illumina Nextera R1, UMI, and the 10x Barcode. The cell barcoded molecules were then cleaned up with Silane DynaBeads and amplified using 14 PCR cycles. Size selection using SPRIselect reagent was performed post amplification to separate full-length cDNA from FBs. Next, full-length, barcoded cDNA was then enzymatically fragmented, sized-selected, adapter-ligated, and amplified for library construction. During the library construction, P5, P7, i7 and i5 sample indexes, and TruSeq Read 2 were added. Separately, FBs were prepared into library constructs by incorporating P5, P7, i7 and i5 sample indexes, and TruSeq Read 2 via PCR. Samples were pooled and run on the NovaSeq 6000 sequencer with a S4 flow cell using the following sequencing parameters: R1: 28 cycles, i7: 10 cycles, i5: 10 cycles, R2: 90 cycles. Sequencing data processing and sample demultiplexing was done using cellranger multi[30]. Matrices were processed with Seurat using standard normalization procedures[31]. Only cells with less than or equal to 5% mitochondrial gene content and number of genes between 500 and 7,500 were kept. Clustering of cells based on graphical features was done using the provided Louvain algorithm. We used multiple resolutions to find the cluster structure that best captures the expected group of hematopoietic cells. Clusters were named based on the expression of lineage-specific genes (markers) using a single-cell atlas of mouse hematopoiesis[32]. Differentially expressed genes between clusters were found with the DESeq2 method[33]. An average expression of each gene from all cells in each cluster was calculated for this purpose.

Whole Exome Sequencing on mouse bone marrow

DNA was isolated from BM mononuclear cells using the Zymo Quick-DNA kit. Mouse Exome was enriched using Twist Mouse Exome probe set following manufacturer's protocols (Twist Biosciences). Samples were sequenced on an Illuumina NovaSeq. FASTQ files were aligned to mouse mm10/GRCm38 genome. Variants were called with DeepVariant and variant annotations with snpEff[34,35].

Antibodies

Antibody	Company	Catalog number	Notes
CD45R (B220) Monoclonal Antibody (RA3-6B2), APC	eBioscience	17-0452-82	
CD11b Monoclonal Antibody (M1/70), PE-Cyanine7	eBioscience	25-0112-81	
Ly-6G/Ly-6C Monoclonal Antibody (RB6-8C5), eFluor™ 450	eBioscience	48-5931-82	
CD45.2 Monoclonal Antibody (104), APC-eFluor™ 780	eBioscience	47-0454-82	
CD45.1 Monoclonal Antibody (A20), PE	eBioscience	12-0453-82	
Brilliant Violet 510™ anti-mouse CD45.1 Antibody	Biolegend	110741	
CD117 (c-Kit) Monoclonal Antibody (2B8), APC	eBioscience	17-1171-81	
CD117 (c-Kit) Monoclonal Antibody (2B8), APC-eFluor™ 780	eBioscience	47-1171-82	
Ly-6A/E (Sca-1) Monoclonal Antibody (D7), PE	eBioscience	12-5981-82	
Ly-6A/E (Sca-1) Monoclonal Antibody (D7), PE-Cyanine7	eBioscience	25-5981-82	
CD135 (Flt3) Monoclonal Antibody (A2F10), APC	eBioscience	17-1351-80	
TER-119 Monoclonal Antibody (TER-119), FITC	ebioscience	11-5921-82	
Anti-Human CD71 (Transferrin Receptor) Antibody, Clone OKT9, APC	Stemcell	60106AZ.1	
CD71 (Transferrin Receptor) Monoclonal Antibody (R17217 (RI7 217.1.4)), PE,	ebioscience	12-0711-81	
CD3e Monoclonal Antibody (145-2C11), PE	eBioscience	12-0031-82	
BD Pharmingen™ FITC Hamster Anti-Mouse CD3e	BD Pharm	553062	
CD48 Monoclonal Antibody (HM48-1), FITC	eBioscience	11-0481-85	
BD Horizon™ BV510 Hamster Anti-Mouse CD48	BD Bioscience	563536	
Streptavidin APC-eFluor™ 780 Conjugate	Thermo Fisher	47-4317-82	
PE/Cy7 anti-mouse CD150 (SLAM) Antibody	Biolegend	115914	
DDX41 Antibody (2F4)	Novus	H00051428-M01	
p53 Antibody (DO-1)	Santa cruz	sc-126	For human cells
p53 (1C12) Mouse mAb	Cell Signaling	2524	For mouse cells
Vinculin (E1E9V) XP® Rabbit mAb	Cell Signaling	13901	

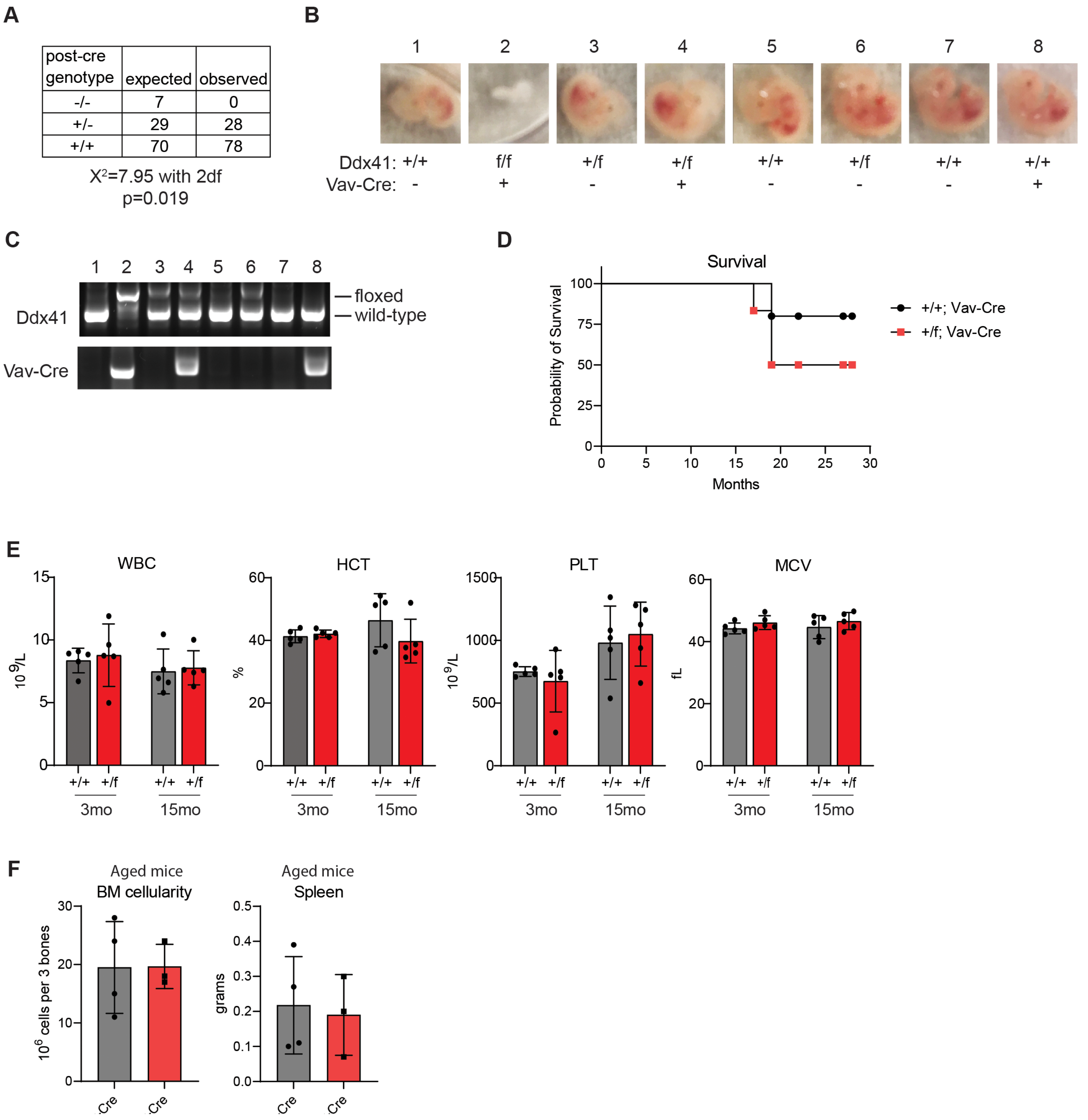

Supplementary Figure 1. Mice with heterozygous loss of Ddx41 in the hematopoietic system are viable with mild reduction in red blood cells over the course of life. (A) Table of observed genotypes of viable pups from Ddx41^{flox} Vav-Cre litters compared to expected Mendelian ratios. Chi-square test indicates a significant difference from expected for the number of Ddx41$^{f/f}$;Vav-Cre+ pups. **(B)** Pictures of E12.5 embryos from a single litter with indicated genotyping result. **(C)** PCR result for genotyping of pups in B. **(D)** Kaplan-Meier plot for survival of mice of indicated genotypes. Mice were age/gender matched. **(E)** Common blood count results on peripheral blood of mice at 3 months and 15 months of age. **(F)** Bone marrow cellularity and spleen weight in aged mice.

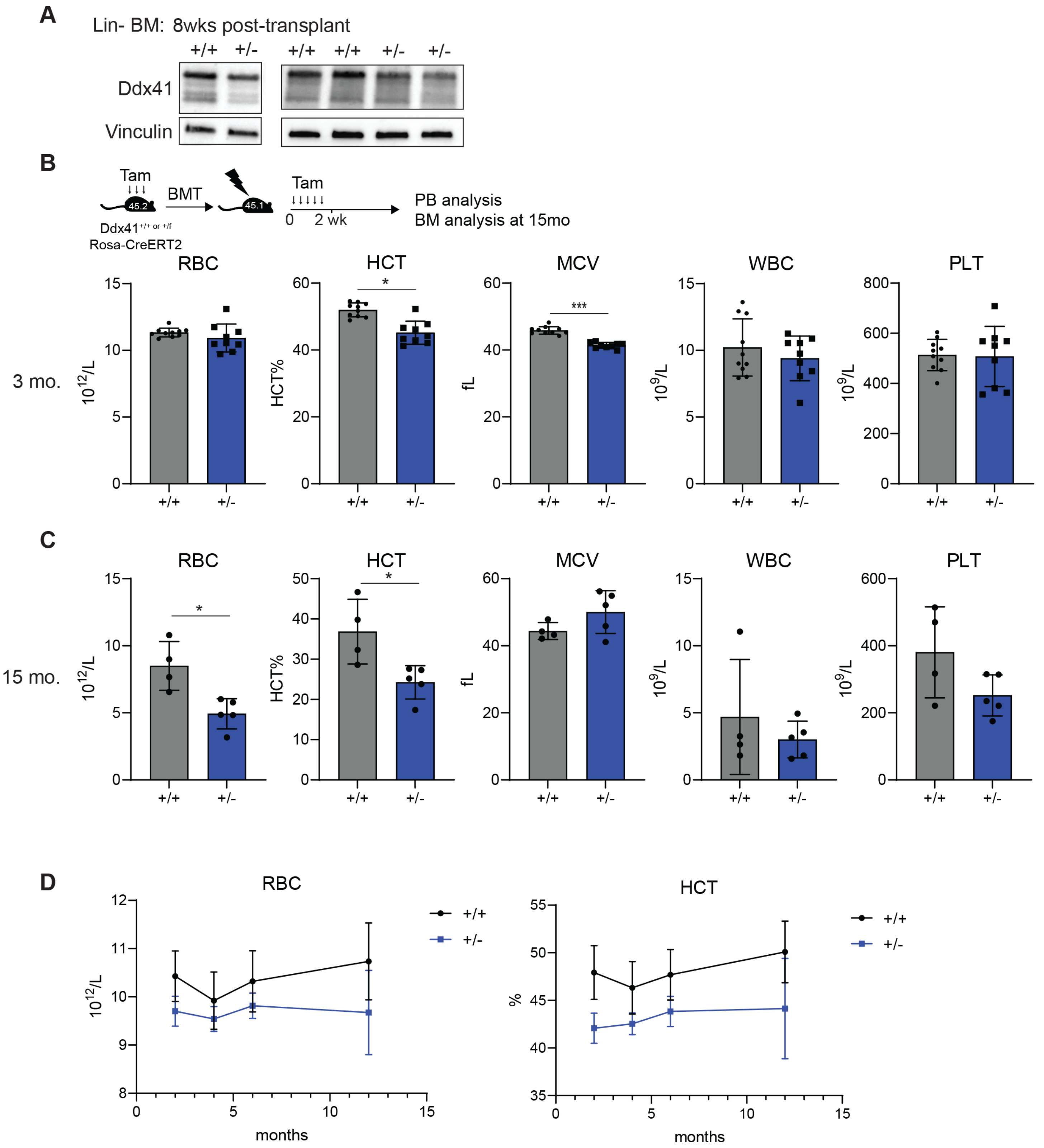

Supplementary Figure 2. Loss of one allele of Ddx41 in hematopoietic cells causes mild reduction in red blood cells over the course of life. (A) Immunoblot on lysates from lineage-negative bone marrow cells with the indicated genotypes harvested 8-weeks post-transplant. **(B)** Common blood count data from mice transplanted with Ddx41$^{+/f}$;Rosa-CreERT2 or control bone marrow and treated with tamoxifen immediately following transplant. Data is from bleeds at 3 months post-transplant. **(C)** Common blood count data from bleeds at 15 months post-transplant. **(D)** Red blood cell number and hematocrit in common blood count analysis of mice transplanted with bone marrow of the indicated genotype.

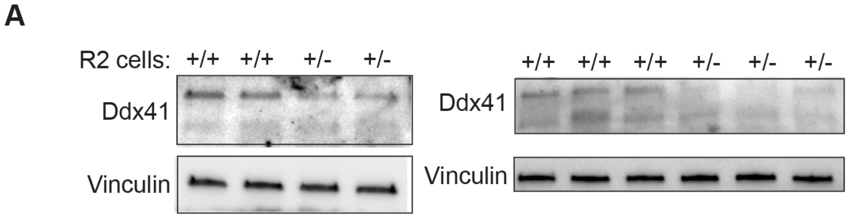

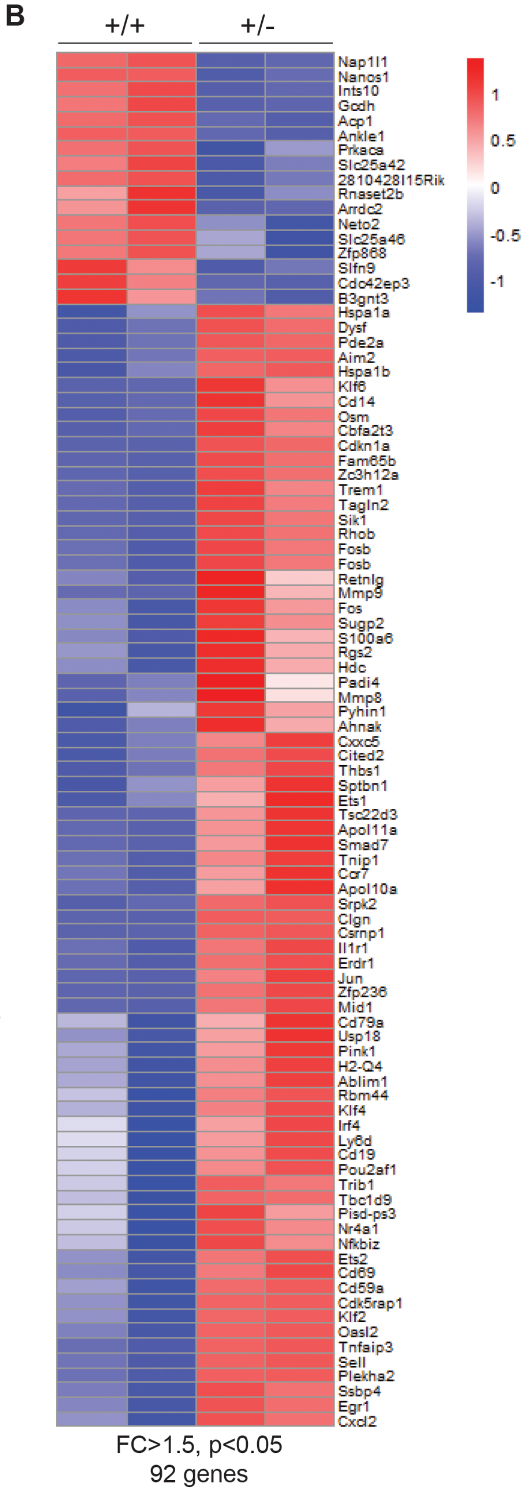

C

Gene Set	pValue
Adaptive Immune System	0.001
Hemostasis	0.00101
Signaling by GPCR	0.00103
G alpha (i) signalling events	0.0012
Amino acid and oligopeptide SLC transporters	0.00131
Interferon Signaling	0.00217
Interferon gamma signaling	0.00227
Cytokine Signaling in Immune system	0.00516
Ribosomal scanning and start codon recognitior	0.00667
Translation initiation complex formation	0.00667

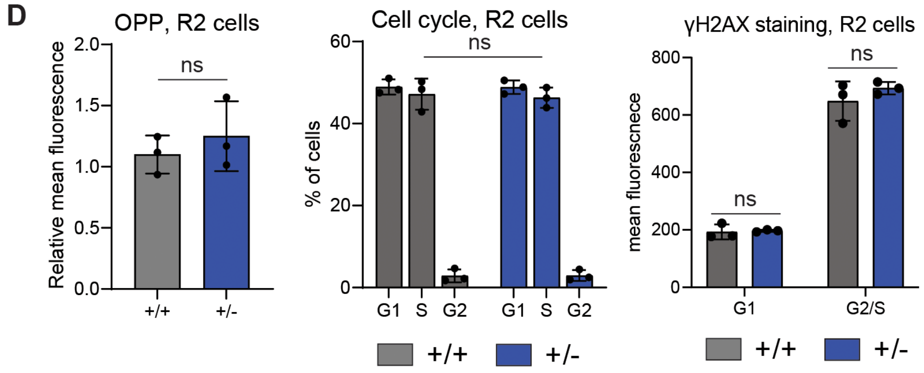

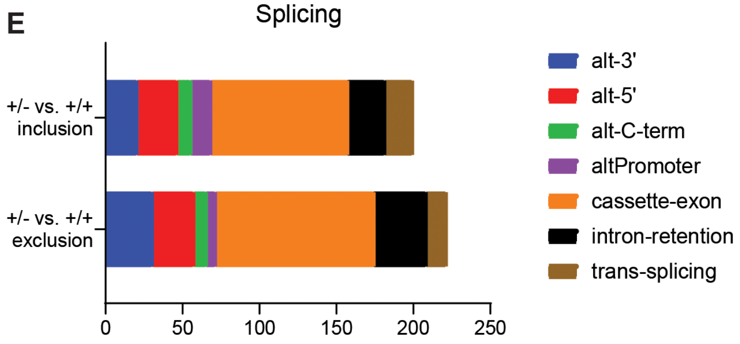

Supplementary Figure 3. Ddx41$^{+/-}$ erythroid progenitors have cell growth defect ex vivo and activated cell stress-related gene expression in vivo. (A) Immunoblot for DDX41 expression in CD71+ Ter119+ erythroid progenitors sorted from Ddx41$^{+/f}$;RosaCreERT2 transplanted bone marrow. **(B)** Heatmap of differentially-expressed genes in CD71+ Ter119+ sorted from transplanted Ddx41$^{+/f}$;RosaCreERT2 bone marrow. **(C)** Gene set enrichment analysis on RNA-Seq data shown in B. **(D)** OP-Puro and EdU incorporation in CD71+ Ter119+ cells in Ddx41$^{+/f}$;RosaCreERT2 transplanted bone marrow. **(E)** Analysis of differential splicing in RNA-Seq data shown in B.

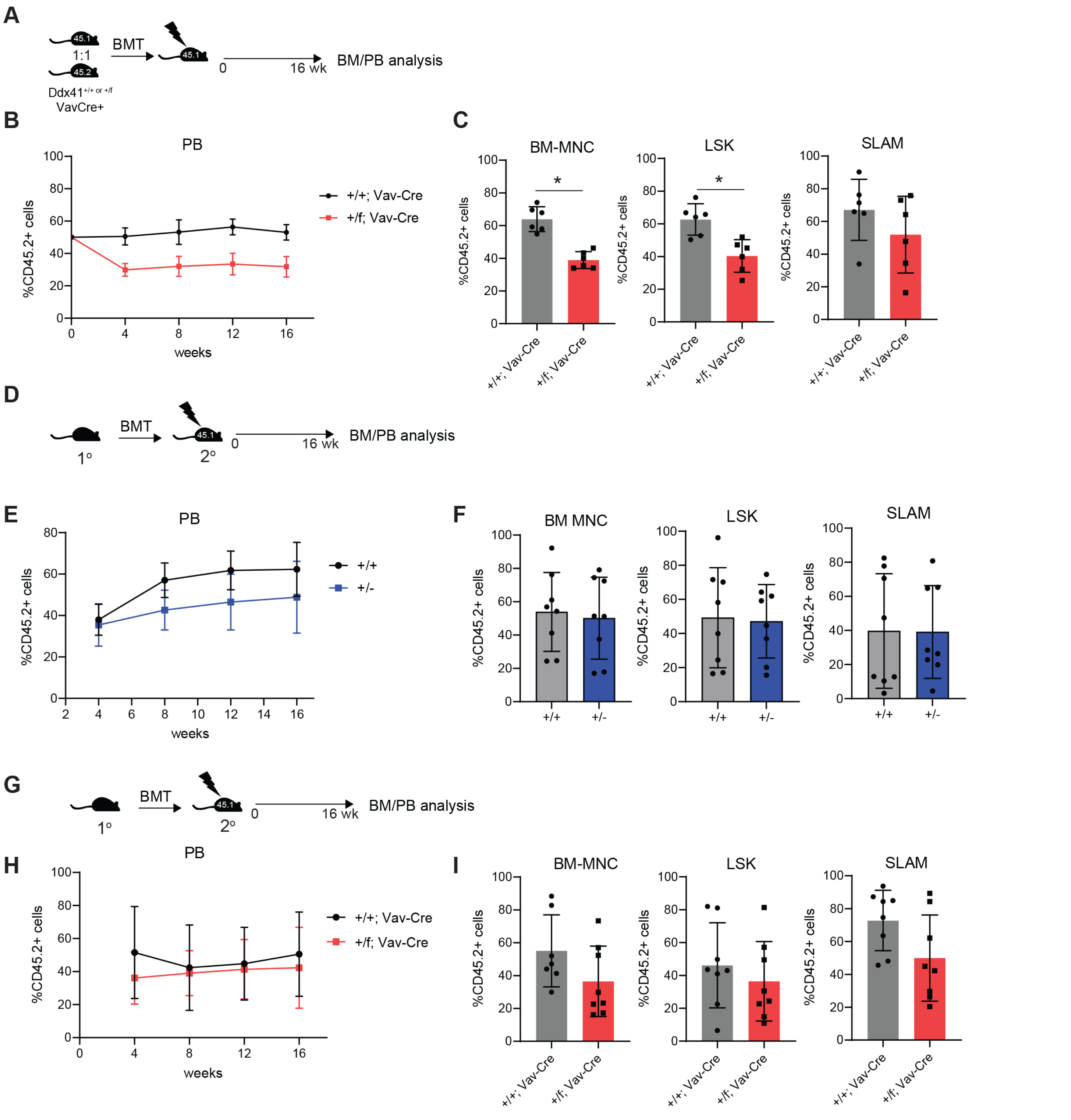

Supplementary Figure 4. Ddx41$^{+/-}$ hematopoietic stem and progenitor cells have modestly reduced fitness, correlating with reduced protein translation activity. (A) Experimental strategy for competitive transplantation of Ddx41$^{+/f}$;Vav-Cre bone marrow mononuclear cells into lethally-irradiated CD45.1+ recipients. **(B)** Percent of CD45.2+ cells in the peripheral blood of transplanted mice from A. **(C)** Percent of CD45.2+ cells in bone marrow mononuclear cells (BM-MNC), LSK (Lin$^-$, Sca-1+, Kit$^+$), LSK-SLAM (LSK, CD48$^-$, CD150$^+$) of transplanted mice from A. **(D)** Experimental strategy for secondary transplant of Ddx41$^{+/f}$;RosaCreERT2 bone marrow cells into lethally-irradiated CD45.1+ recipients. **(E)** Percent of CD45.2+ cells in the peripheral blood of transplanted mice from D. **(F)** Percent of CD45.2+ cells in bone marrow mononuclear cells (BM-MNC), LSK (Lin$^-$, Sca-1+, Kit$^+$), LSK-SLAM (LSK, CD48$^-$, CD150$^+$) of transplanted mice from D. **(G)** Experimental strategy for secondary transplant of Ddx41$^{+/f}$;VavCre bone marrow cells into lethally-irradiated CD45.1+ recipients. **(H)** Percent of CD45.2+ cells in the peripheral blood of transplanted mice from G. **(I)** Percent of CD45.2+ cells in

bone marrow mononuclear cells, LSK (Lin^-, Sca-1+, Kit^+), LSK-SLAM (LSK, $CD48^-$, $CD150^+$) of transplanted mice from G.

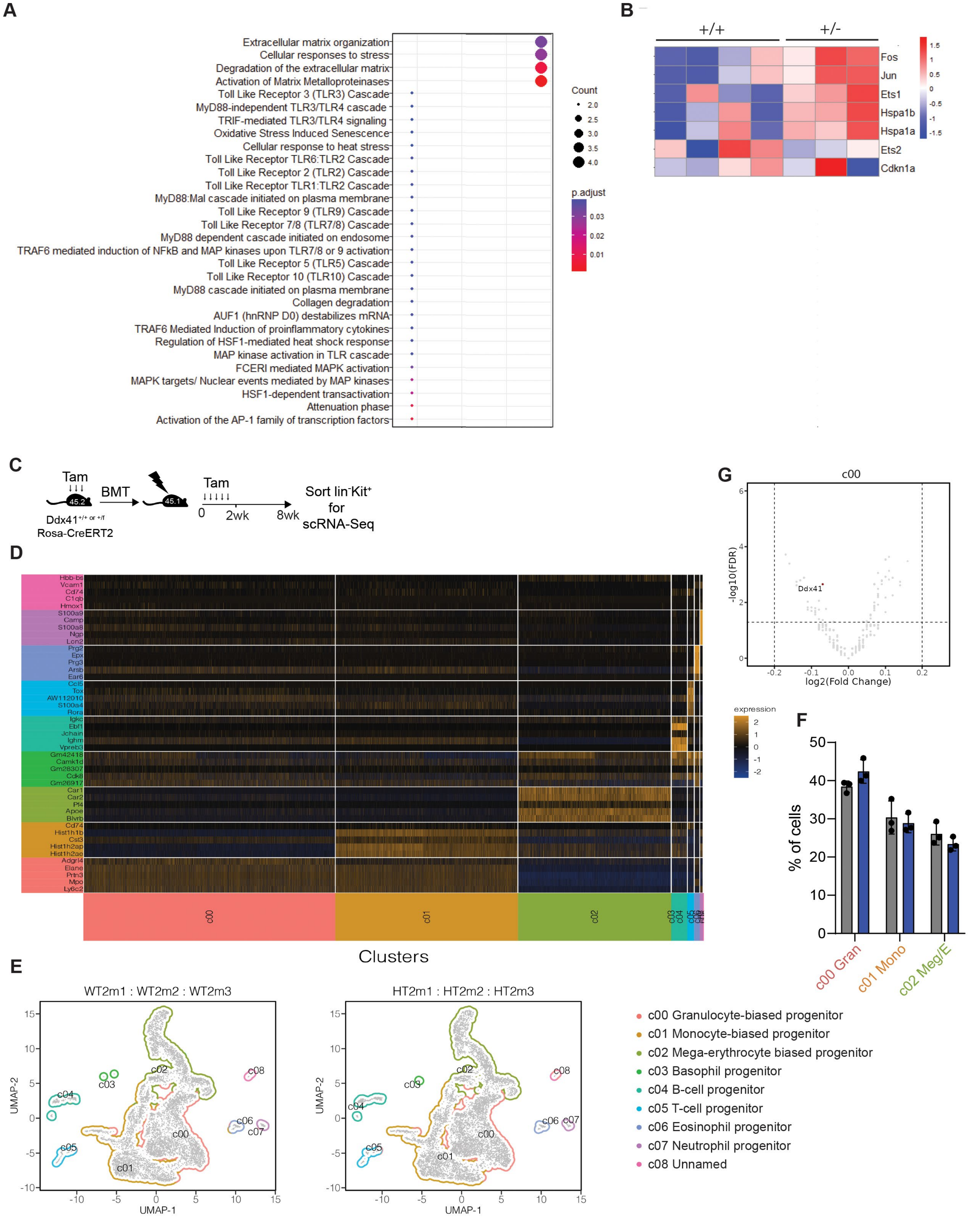

Supplementary Figure 5. Transcriptome analysis in Ddx41$^{+/-}$ hematopoietic stem and progenitor cells indicates activation of cellular stress pathways. (A) Over-representation analysis for REACTOME pathways on differentially-expressed genes from RNA-Seq on LSK cells. **(B)** Heatmap for expression of genes from

“Cellular response to stress” REACTOME pathway in RNA-Seq from A. **(C)** Experimental setup and tamoxifen-injection schedule for scRNA-Seq on Lin^-Kit^+ cells from non-competitive transplant mice. **(D)** Heatmap depicting the expression of key genes used to identify clusters in single-cell RNA Seq data from $Ddx41^{+/f}$;RosaCreRT2 Lin^- bone marrow cells 8-weeks post-tamoxifen treatment **(E)** Clustering of cells from scRNA-Seq and naming of each cluster based on expression of lineage-specific genes. **(F)** Percentage of cells in the indicated populations as determined by scRNA-Seq. **(G)** Volcano plot of differential gene expression in scRNA-Seq data from cluster c00.

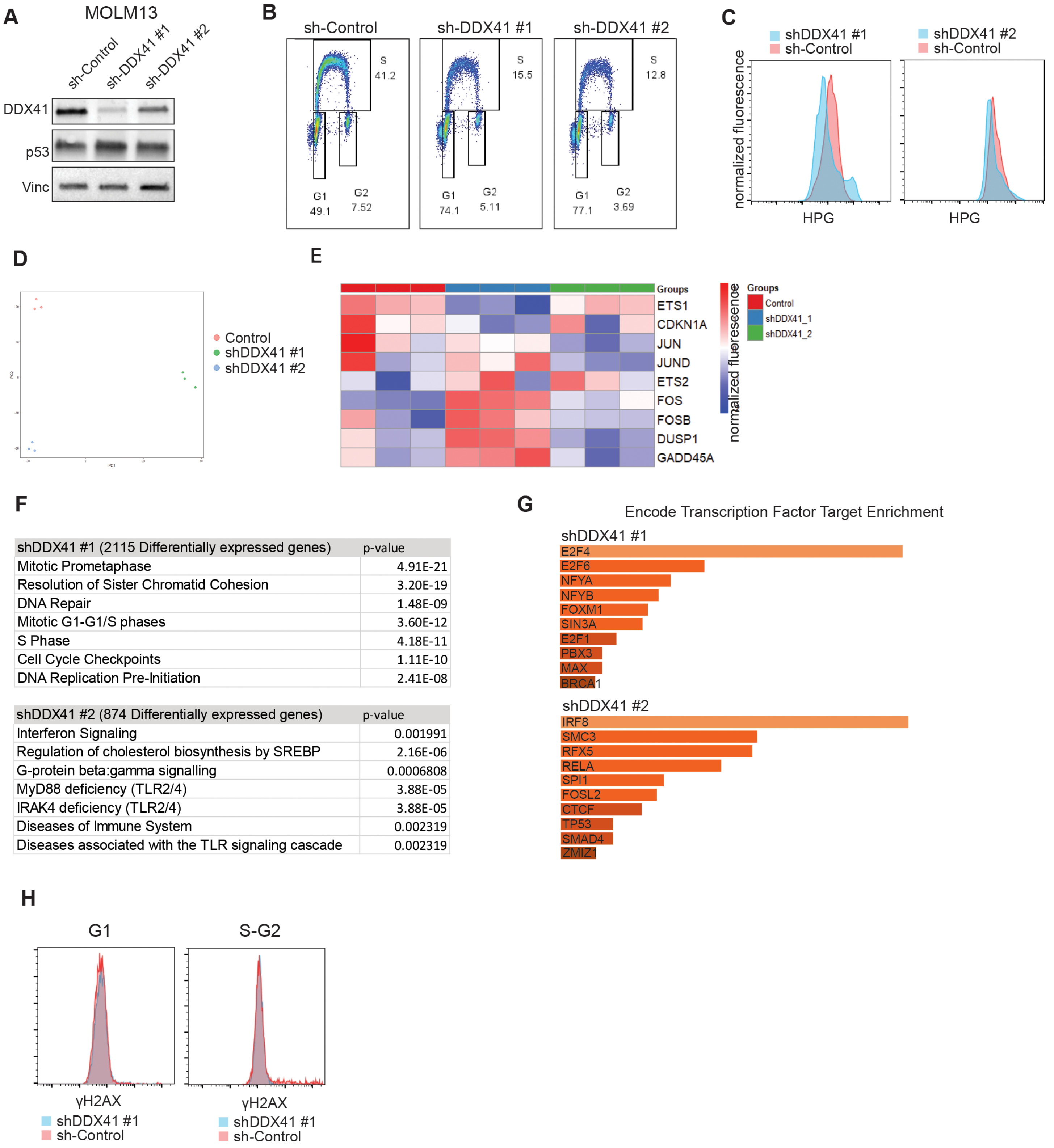

shDDX41 #1 (2115 Differentially expressed genes)	p-value
Mitotic Prometaphase	4.91E-21
Resolution of Sister Chromatid Cohesion	3.20E-19
DNA Repair	1.48E-09
Mitotic G1-G1/S phases	3.60E-12
S Phase	4.18E-11
Cell Cycle Checkpoints	1.11E-10
DNA Replication Pre-Initiation	2.41E-08

shDDX41 #2 (874 Differentially expressed genes)	p-value
Interferon Signaling	0.001991
Regulation of cholesterol biosynthesis by SREBP	2.16E-06
G-protein beta:gamma signalling	0.0006808
MyD88 deficiency (TLR2/4)	3.88E-05
IRAK4 deficiency (TLR2/4)	3.88E-05
Diseases of Immune System	0.002319
Diseases associated with the TLR signaling cascade	0.002319

Supplementary Figure 6. Loss of DDX41 in MOLM13 cells causes p53 signaling in the absence of DNA damage. (A) Western blot for DDX41 and p53 expression in MOLM13 cells expressing anti-DDX41 shRNAs. **(B)** Representative flow plots for cell cycle by EdU incorporation in cultures of MOLM13 cells expressing anti-DDX41 shRNAs. **(C)** Representative flow plots for HPG incorporation in MOLM13 cells expressing anti-DDX41 shRNAs compared to sh-control. **(D)** Principle component analysis on RNA-Seq samples for MOLM13 cells

transduced with shRNAs targeting DDX41 compared to control shRNA. **(E)** Heatmap for expression of genes from “Cellular response to stress” REACTOME pathway in MOLM13 RNA-Seq. **(F)** Over-representation analysis for REACTOME pathways on differentially-expressed genes from MOLM13 RNA-Seq. **(G)** Analysis of enrichment for target genes of transcription factors using ENCODE ChIP-Seq data. **(H)** Representative flow plots for γH2AX staining in MOLM13 cells. DAPI incorporation is utilized to distinguish cells in G1 vs. S-G2 phase of the cell cycle.

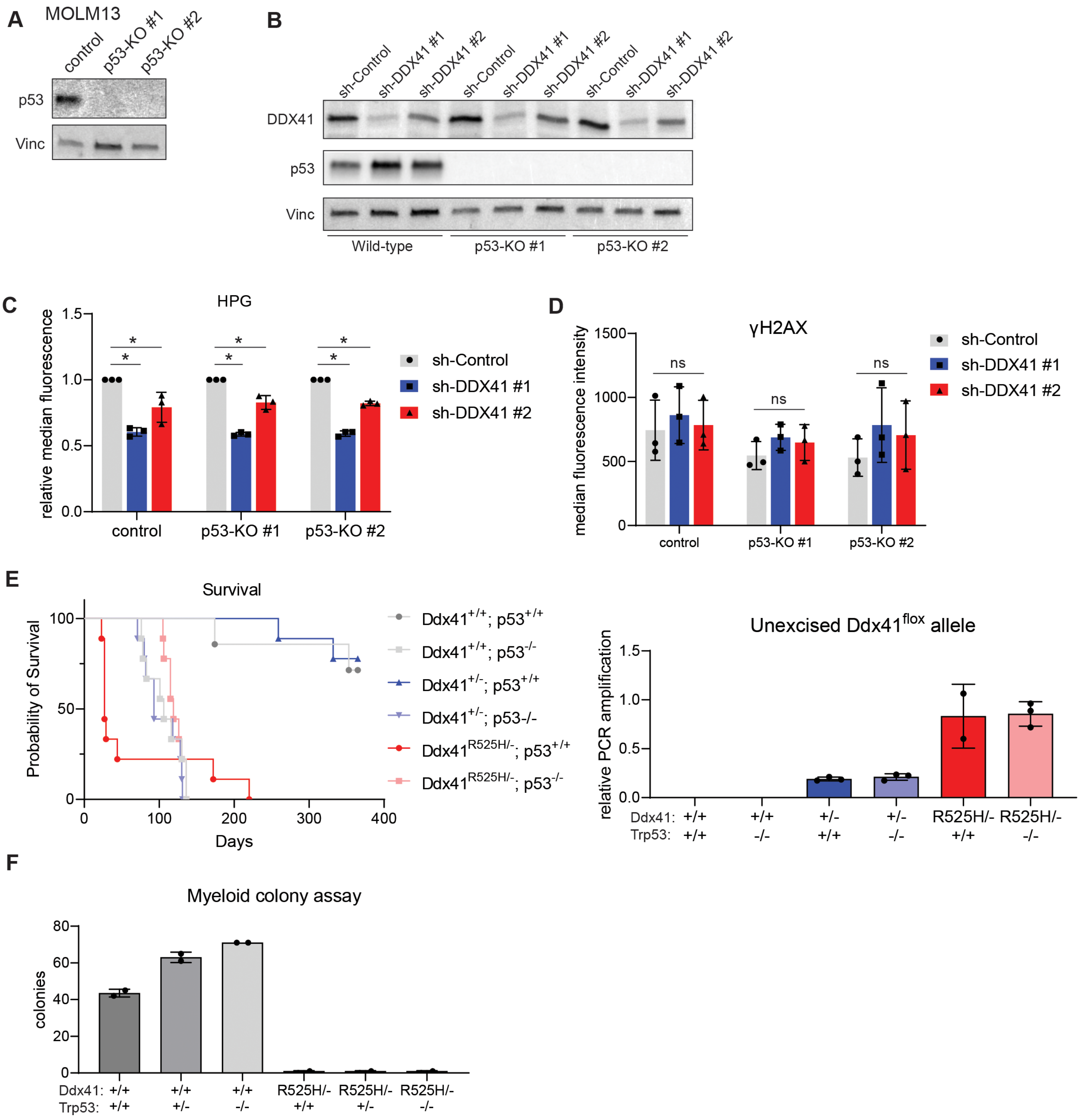

Supplementary Figure 7. Knockout of p53 confers partial rescue of cell growth defects caused by partial but not complete loss of DDX41. **(A)** Western blot for p53 expression in p53-knockout MOLM13 cells generated by CRISPR. **(B)** Western blot for DDX41 and p53 expression in p53-knockout MOLM13 cells expressing anti-DDX41 shRNAs. **(C)** Measurement of protein translation activity by flow cytometry for HPG incorporation in p53-knockout MOLM13 cells expressing anti-DDX41 shRNAs compared to sh-control. **(D)** Measurement of DNA damage by flow cytometry for γH2AX in p53-knockout MOLM13 cells expressing anti-DDX41 shRNAs compared to sh-control. **(E)** Kaplan-Meier curve for survival of mice transplanted with bone marrow cells of the indicated genotype. Floxed or conditional Ddx41 alleles were excised by activation of RosaCreERT2 by tamoxifen injection beginning 3-days post-transplant. (Right panel) Quantification of unexcised Ddx41^{flox} alleles by real-time PCR with primers recognizing the floxed portion of the conditional Ddx41 gene. This analysis indicates that the

surviving bone marrow cells in Ddx41$^{R525H/-}$ mice have unexcised Ddx41 alleles due to strong selective pressure against excision. **(F)** Colony assay on Lin^{-} bone marrow cells from RosaCretERT2+ mice of the indicated genotype. Ddx41 alleles were excised by activation of RosaCreERT2 by addition of 4-OH-tamoxifen to the semisolid media.

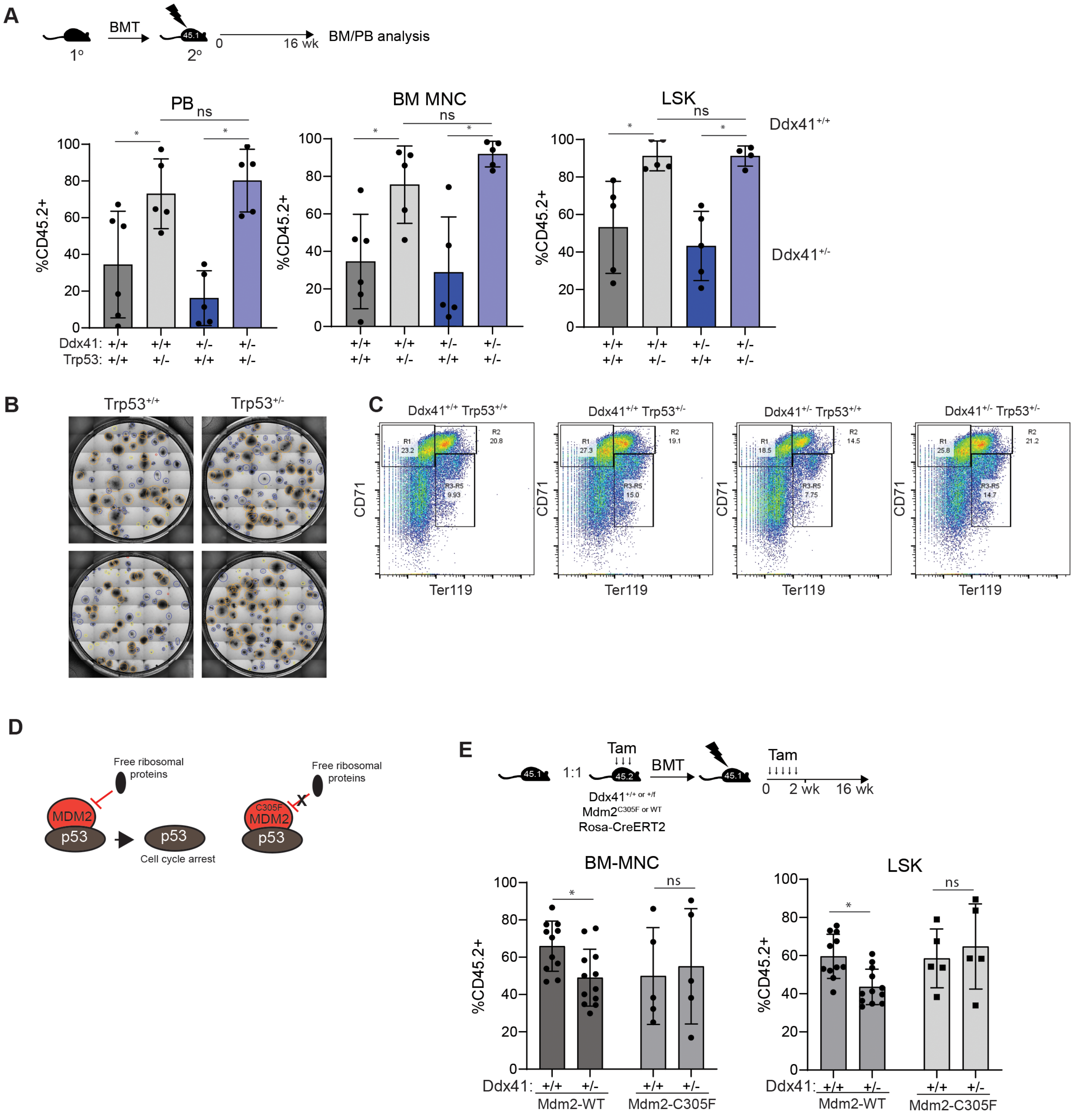

Supplementary Figure 8. Heterozygous loss of p53 rescues reduced fitness phenotype of Ddx41$^{+/-}$ hematopoietic stem and progenitor cells. (A) Secondary transplant of Ddx41$^{+/-}$;p53$^{+/-}$ bone marrow cells into lethally-irradiated CD45.1+ recipients. The percent engraftment is determined by percentage of CD45.2+ cells in the peripheral blood, bone marrow, and LSK populations. **(B)** Representative image of colony formation in methylcellulose from LSK cells sorted from non-competitive bone marrow transplant mice at 8wks post-transplant. **(C)** Representative flow plots for in vitro erythroid differentiation cultures of Lin^{-} bone marrow cells from Ddx41^{f};p53$^{+/-}$;RosaCreERT2 cells. **(D)** Schematic depicting the effect of the MDM2-C305F mutation on the regulation of p53 abundance by free ribosomal proteins. **(E)** Competitive transplant for the effect of the Mdm2^{C305F} mutation on hematopoietic stem and progenitor cell function in Ddx41$^{+/f}$;Rosa-CreERT2 donor mice. Chimerism of CD45.2+ cells in recipients is measured by flow cytometry on bone marrow mononuclear cells.

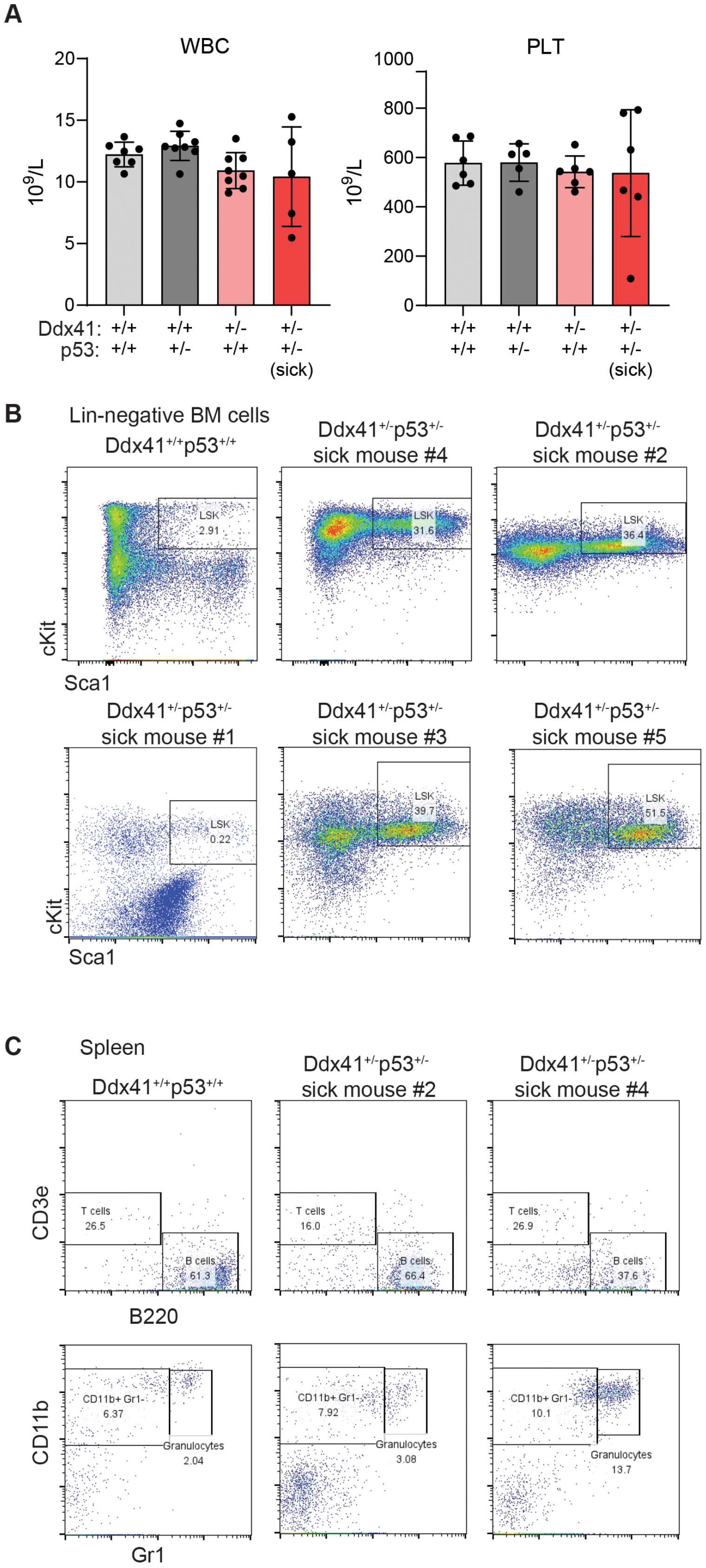

Supplementary Figure 9. Combined heterozygosity of Ddx41 and p53 causes near-fully penetrant hematologic malignancy. (A) Common blood count results at end of experiment (12 months) or point of sacrifice on peripheral blood of mice transplanted with bone marrow cells of the indicated genotype **(B)** Flow cytometry depicting the proportion of Lin^- bone marrow cells residing in the LSK gate in sick mice compared to a healthy control mouse. **(C)** Flow cytometry for the proportion of lymphoid and myeloid cells in the spleen of sick mice compared to a healthy control.

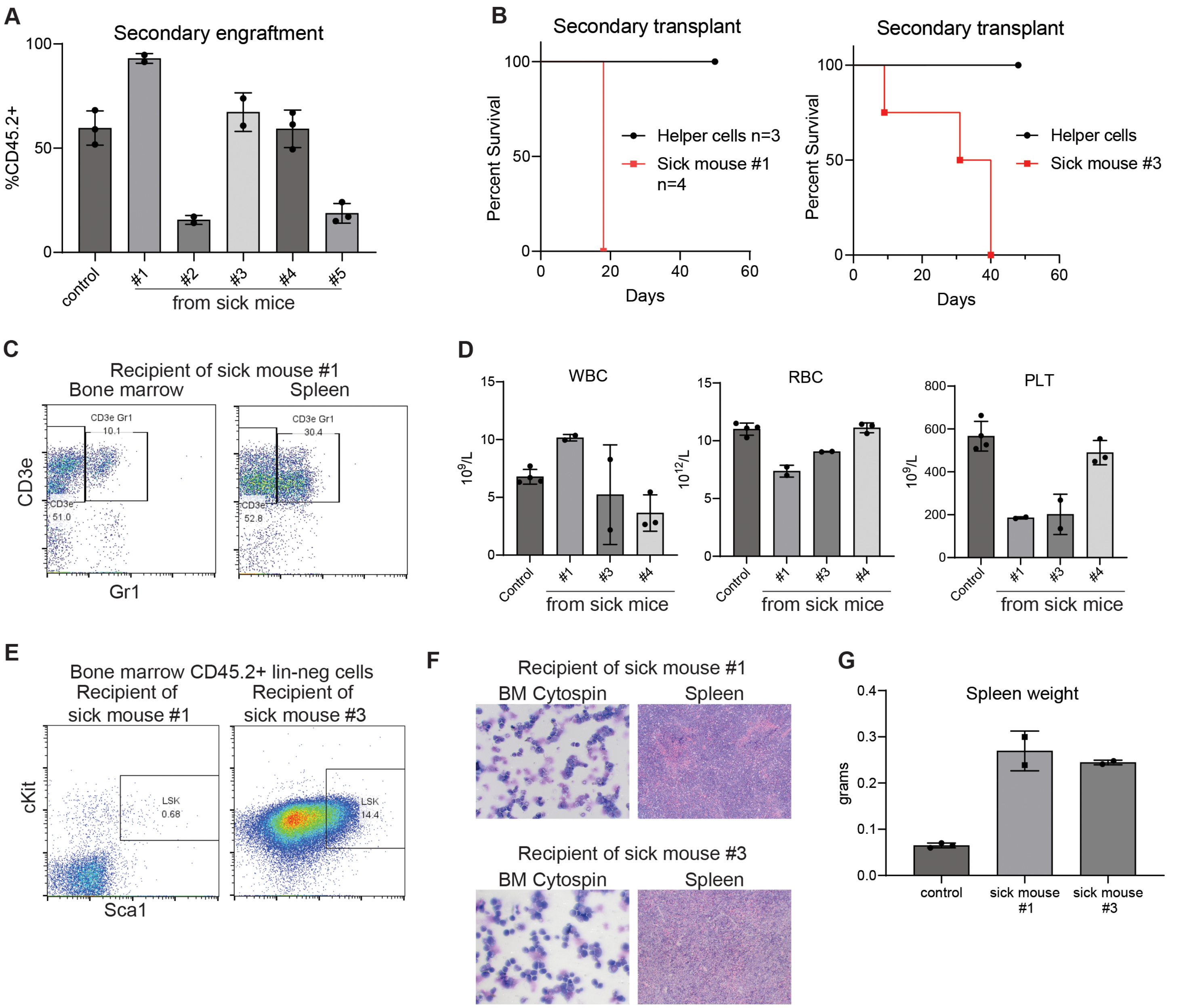

Supplementary Figure 10. Malignant cells from Ddx41$^{+/-}$; p53$^{+/-}$ bone marrows are transplantable to secondary recipients in some cases. (A) Percent CD45.2+ chimerism in the bone marrow of mice transplanted with cells from sick mice. **(B)** Kaplan-Meier plot for survival of mice transplanted with bone marrow from sick mouse #1 and #3. **(C)** Flow cytometry for mixed-lineage leukemia cells in the bone marrow and spleen of mice transplanted with cells from sick mouse #1. **(D)** Common blood count data for transplant recipients of bone marrow from sick mice where engraftment occurred. **(E)** Flow cytometry for CD45.2+ LSK cells in the bone marrow of mice transplanted with cells from sick mice #1 and #3. **(F)** Wright-Geimsa staining of bone marrow cytospins and H&E staining of spleen sections from recipient mice. **(G)** Spleen weights of recipient mice at time of sacrifice.
